# Age and Diet Affect Genetically Separable Secondary Injuries that Cause Acute Mortality Following Traumatic Brain Injury in *Drosophila*

**DOI:** 10.1534/g3.116.036194

**Published:** 2016-10-17

**Authors:** Rebeccah J. Katzenberger, Barry Ganetzky, David A. Wassarman

**Affiliations:** * Department of Medical Genetics, School of Medicine and Public Health, University of Wisconsin–Madison, Wisconsin 53706; † Department of Genetics, College of Agriculture and Life Sciences, University of Wisconsin–Madison, Wisconsin 53706

**Keywords:** *Drosophila* Genetic Reference Panel, gene expression, hyperglycemia innate immune response, repetitive TBI, RNA-seq

## Abstract

Outcomes of traumatic brain injury (TBI) vary because of differences in primary and secondary injuries. Primary injuries occur at the time of a traumatic event, whereas secondary injuries occur later as a result of cellular and molecular events activated in the brain and other tissues by primary injuries. We used a *Drosophila melanogaster* TBI model to investigate secondary injuries that cause acute mortality. By analyzing mortality percentage within 24 hr of primary injuries, we previously found that age at the time of primary injuries and diet afterward affect the severity of secondary injuries. Here, we show that secondary injuries peaked in activity 1–8 hr after primary injuries. Additionally, we demonstrate that age and diet activated distinct secondary injuries in a genotype-specific manner, and that concurrent activation of age- and diet-regulated secondary injuries synergistically increased mortality. To identify genes involved in secondary injuries that cause mortality, we compared genome-wide mRNA expression profiles of uninjured and injured flies under age and diet conditions that had different mortalities. During the peak period of secondary injuries, innate immune response genes were the predominant class of genes that changed expression. Furthermore, age and diet affected the magnitude of the change in expression of some innate immune response genes, suggesting roles for these genes in inhibiting secondary injuries that cause mortality. Our results indicate that the complexity of TBI outcomes is due in part to distinct, genetically controlled, age- and diet-regulated mechanisms that promote secondary injuries and that involve a subset of innate immune response genes.

Traumatic brain injury (TBI) is characterized by a broad spectrum of physical, cognitive, emotional, and behavioral impairments that are caused by primary and secondary injuries (Masel and Dewitt 2010; Brooks *et al.* 2013; Smith *et al.* 2013; Stocchetti and Zanier 2016). Primary injuries result from direct mechanical forces to the brain that occur at the time of a traumatic event, whereas secondary injuries result from cellular and molecular mechanisms subsequently triggered in the brain and other tissues by primary injuries. TBI outcomes differ among individuals not only because they receive different primary injuries but also because their responses produce different secondary injuries (Wang *et al.* 2014; Krishnamurthy and Laskowitz 2016). Therefore, a better understanding of secondary injury mechanisms and the connection between secondary injuries and outcomes is essential to advance the diagnosis and treatment of TBI.

In humans, age at the time of primary injuries and diet immediately after injuries strongly impact resulting secondary injuries (Susman *et al.* 2002; Wang *et al.* 2013; Mychasiuk *et al.* 2015). Among individuals that sustain similar primary injuries, older individuals have a higher probability of mortality than younger individuals, suggesting that biological processes that change during aging promote secondary injuries (Hukkelhoven *et al.* 2003; Dhandapani *et al.* 2012). Also, in a rat TBI model, fasting compared with feeding *ad libitum* following primary injuries is neuroprotective, suggesting that dietary intake enhances biological processes that promote secondary injuries (Davis *et al.* 2008). The innate immune response may be a relevant biological process because it is regulated by age and diet in uninjured animals, and it plays an important role in determining the extent of brain injury in animals that sustain primary injuries (Rivest 2009; Woodcock and Morganti-Kossmann 2013; Djordjevic *et al.* 2016). Immediately following primary injuries, damage-associated molecular patterns (DAMPs), such as various intracellular proteins, are rapidly released into the extracellular space and activate Toll-like receptors (TLRs), which play a key role in the innate immune response (Heiman *et al.* 2014; Gadani *et al.* 2015). Activation of TLRs leads to secretion of inflammatory mediators, such as pro- and anti-inflammatory cytokines, chemokines, complement factors, and reactive oxygen species (ROS). Secreted inflammatory mediators can have either beneficial or detrimental effects, depending on the extent, time, and site of induction (Hellewell and Morganti-Kossmann 2012). However, the mechanisms underlying the beneficial and detrimental effects and the induction parameters are not yet clearly defined.

To investigate secondary injury mechanisms, we used a *Drosophila melanogaster* TBI model that we previously developed (Katzenberger *et al.* 2013, 2015b). The fly TBI model uses a spring-based High-Impact Trauma (HIT) device to inflict mechanical injuries. When the spring with an attached vial of flies is pulled back and released, the vial strikes a polyurethane pad and mechanical forces are delivered to flies as they contact the vial wall. An immediate outcome is temporary incapacitation, indicating that the HIT device delivers primary injuries to the brain (Katzenberger *et al.* 2013, 2015a). Furthermore, injuries to the brain are indicated by outcomes that are shared with rodent TBI models that deliver primary injuries exclusively to the brain. Shared outcomes include increased permeability of the blood–brain barrier and intestine, as well as neurodegeneration in the brain (Feighery *et al.* 2008; Bansal *et al.* 2009; Katzenberger *et al.* 2013, 2015a, 2015c; Smith *et al.* 2013; Alluri *et al.* 2015).

In the fly TBI model, mortality percentage within 24 hr of primary injuries, termed the Mortality Index at 24 hr (MI_24_), is affected by both primary and secondary injuries. The MI_24_ increases as the severity of primary injuries increases, indicating a role for primary injuries in determining the MI_24_ (Katzenberger *et al.* 2015b). Furthermore, under conditions where primary injuries are held constant, the MI_24_ is affected by age at the time of primary injuries and diet following primary injuries, indicating roles for secondary injuries in determining the MI_24_ (Katzenberger *et al.* 2013, 2015a). The MI_24_ is closely correlated with increased intestinal permeability, suggesting that factors that leak from the intestine cause death following TBI (Katzenberger *et al.* 2015a). Bacteria leak from the intestine into the hemolymph and activate the innate immune response, as determined by mRNA levels of antimicrobial peptide (AMP) genes that are transcriptional targets of the Toll- and Immune-deficient (Imd) innate immune response pathways (Lemaitre *et al.* 1997; Lemaitre and Hoffmann 2007; Katzenberger *et al.* 2015a). Increased expression of some AMPs following TBI is significantly diminished in flies that lack bacteria, but the MI_24_ is not affected, indicating that activation of the innate immune response by endogenous bacteria neither prevents nor promotes mortality (Katzenberger *et al.* 2015a). Nevertheless, flies lacking bacteria still induce expression of AMPs following TBI, suggesting that other factors, such as DAMPs and ROS, activate the innate immune response and may prevent or promote mortality.

In addition to bacteria, glucose leaks from the intestine into the hemolymph following TBI (Katzenberger *et al.* 2015a). Moreover, reducing glucose levels in the hemolymph by feeding flies water rather than molasses food following primary injuries reduces the MI_24_. Hyperglycemia is not only associated with mortality following TBI in flies but also in humans, suggesting that mechanisms underlying hyperglycemia-mediated secondary injuries are evolutionarily conserved (Griesdale *et al.* 2009; Borsage *et al.* 2015; Chong *et al.* 2015).

To identify genes involved in secondary injury pathways, we examined global gene expression following primary injuries and compared these data among age and diet conditions that held primary injuries constant but produced different MI_24_s. We found that expression of innate immune response genes dominated the early transcriptional response to primary injuries, and that expression of some of these genes was affected by age and diet conditions. Our results indicate that the complexity of TBI outcomes is due in part to distinct, genetically controlled, age- and diet-regulated mechanisms that promote secondary injuries and involve a subset of innate immune response genes.

## Materials and Methods

### Fly lines and culturing

*Drosophila* Genetic Reference Panel (DGRP) flies were obtained from the Bloomington Stock Center. The 30 RAL lines that were examined were randomly chosen from the DGRP collection (Mackay *et al.* 2012). Flies were maintained on molasses food at 25° unless otherwise stated. Molasses food contained 30 g Difco granulated agar (Becton-Dickinson, Sparks, MD), 44 g YSC-1 yeast (Sigma, St. Louis, MO), 328 g cornmeal (Lab Scientific, Highlands, NJ), 400 ml unsulfured Grandma’s molasses (Lab Scientific), 3.6 liter water, 40 ml propionic acid (Sigma), and tegosept (8 g methyl 4-hydroxybenzoate in 75 ml of 95% ethanol; Sigma). Water vials were prepared immediately before use by placing a circular piece of Whatman filter paper (GE Healthcare Bio-Sciences, Pittsburgh, PA) at the bottom of the vial to absorb 200 μl of water.

### Behavioral and molecular assays

The HIT device was operated as described in Katzenberger *et al.* (2015b). All experiments were performed with HIT device number 1, the same device used in Katzenberger *et al.* (2013) and Katzenberger *et al.* (2015a). The MI_24_ and longevity were determined as described in Katzenberger *et al.* (2013). Quantitative real-time reverse transcription PCR (RT-qPCR) was performed on total RNA extracted from whole flies as described in Petersen *et al.* (2012). Supplemental Material, Table S5 contains primer sequences used in the RT-qPCR analyses.

### Construction of mRNA libraries and high-throughput sequencing

RNA to generate libraries for RNA sequencing (RNA-seq) was extracted from whole male flies as described in Petersen *et al.* (2012). RNA was extracted from younger (0–7 d old) and older (20–27 d old) flies after receiving four strikes from the HIT device with 5-min interinjury intervals and feeding on water for 2 hr or food for 4 hr. In addition, RNA was extracted from uninjured younger and older flies fed water for 2 hr or food for 4 hr. RNA quality control, library preparation, and sequencing were performed at the University of Wisconsin–Madison Biotechnology Center. Each RNA library was generated following the Illumina TruSeq RNA Sample Preparation v2 (Rev. F) Guide using the Illumina TruSeq RNA Sample Preparation Kit (Illumina Inc., San Diego, CA). mRNA was purified from 1 μg total RNA using polyT oligo-attached magnetic beads. Following purification, mRNA was fragmented using divalent cations under elevated temperature. Double-stranded cDNA was synthesized using SuperScript II Reverse Transcriptase (Invitrogen, Carlsbad, CA) and random primers for first strand cDNA synthesis, followed by second strand synthesis using DNA Polymerase I and RNase H for removal of mRNA. Double-stranded cDNA was purified using Agencourt AMPure XP beads (Qiagen, Valencia, CA), blunt end-repaired by T4 DNA Polymerase and Klenow DNA Polymerase, and phosphorylated by T4 Polynucleotide Kinase. Blunt-ended cDNA was purified using Agencourt AMPure XP beads. cDNA products are incubated with Klenow DNA Polymerase to add an adenine to the 3′ end of the blunt phosphorylated DNA fragments and then purified using Agencourt AMPure XP beads. DNA fragments were ligated to Illumina adapters, which have a single thymine overhang at their 3′ end and then purified using Agencourt AMPure XP beads. Adapter-ligated DNA was amplified by Linker-mediated PCR for 10 cycles using Phusion DNA Polymerase and Illumina PE genomic DNA primer set, followed by purification using Agencourt AMPure XP beads. Quality and quantity of finished libraries were assessed using an Agilent DNA1000 series chip assay (Agilent Technologies, Santa Clara, CA) and Invitrogen Qubit HS Kit (Invitrogen), respectively. Each library was standardized to 2 μM. Cluster generation was performed using a TruSeq Rapid Single Read Cluster Kit (v2) and the Illumina cBot, with libraries multiplexed for 1 × 100 bp sequencing using the TruSeq Rapid SBS kit (v2) on an Illumina HiSeq2500. Images were analyzed using CASAVA (Hosseini *et al.* 2010).

### RNA-seq analysis

Sequencing reads were adapter and quality trimmed using the Skewer trimming program (Jiang *et al.* 2014). Quality reads were subsequently aligned to the *D. melanogaster* genome using the STAR aligner (Dobin *et al.* 2013). Quantification of expression for each gene was calculated by RSEM (Li and Dewey 2011). The expected read counts from RSEM were filtered for low/empty values and used for differential gene expression analysis using edgeR (Robinson *et al.* 2010).

### Data availability

Gene expression data are available in the GEO database under accession number GSE85821. Table S1 contains statistical analyses of data in Figure 3, A and B, which is summarized in Table 1. Table S2 lists genes categorized in Figure 4A that were up-regulated following primary injuries. Table S3 lists genes categorized in Figure 4B that were down-regulated following primary injuries. Table S4 contains data used for analysis of absolute expression after primary injuries that are shown in Figure 6, Figure 7, Figure 8, Figure 9, and Figure 10. Table S5 contains the sequence of primers used for RT-qPCR analysis.

**Table 1:** Percentage of RAL lines with a significantly altered MI_24_ between the indicated conditions (*P* ≤ 0.05)

	Age: Younger *vs.* Older				Diet: Water *vs.* Food				Interinjury Interval: 5 min *vs.* 2 hr			
	Water	Food	Water	Food	Younger	Older	Younger	Older	Younger	Older	Younger	Older
	5 min	5 min	2 hr	2 hr	5 min	5 min	2 hr	2 hr	Water	Water	Food	Food
Increased	57	63	40	60	63	70	57	53	0	0	7	7
Decreased	0	0	0	0	0	0	0	0	0	0	0	10
No change	43	37	60	40	37	30	43	47	100	100	93	83

## Results

### Mortality-associated secondary injuries occur 1–8 hr after primary injuries

Studies of mammalian TBI models suggest that the timing of secondary injuries can be determined by altering the interval between primary injuries. In general, increasing the interinjury interval reduces physical, cognitive, and behavioral sequelae, as well as mortality (Kanayama *et al.* 1996; Meehan *et al.* 2012; Huang *et al.* 2013; Weil *et al.* 2014; Bolton Hall *et al.* 2016). For example, the mortality percentage is significantly lower for piglets subjected to primary injuries 1 wk apart compared with 1 d apart (Friess *et al.* 2009). These data indicate that secondary injury mechanisms are active 1 d after but not 1 wk after an initial primary injury. Thus, to determine the timing of secondary injuries that cause mortality in flies following TBI, we altered the interval between primary injuries.

**Figure 1:**
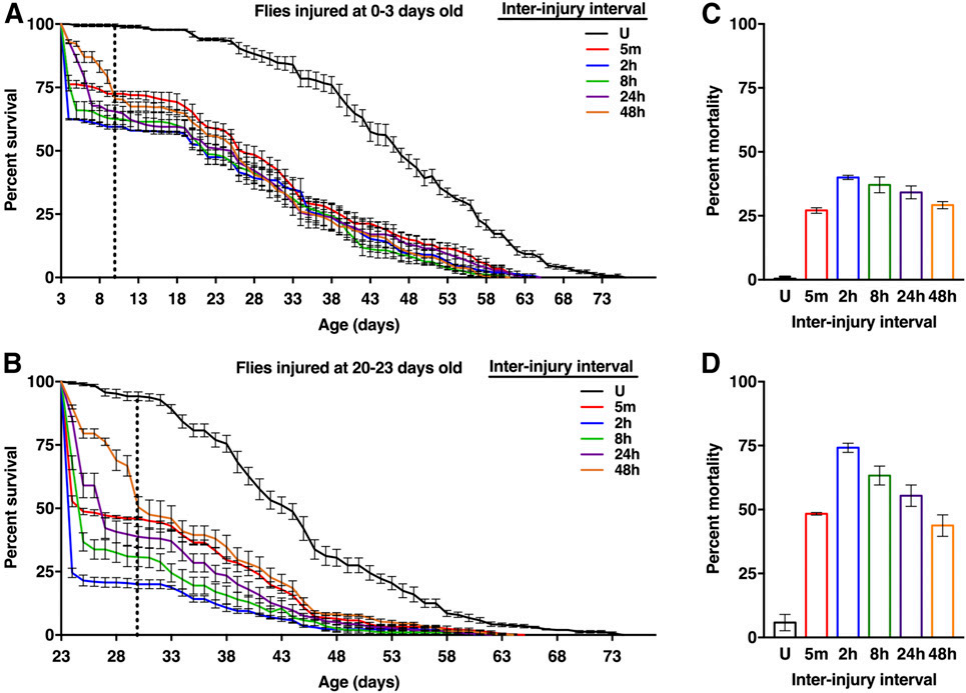
Interinjury interval affects the mortality of *w^1118^* flies. (A and C) 0–3 and (B and D) 20–23 d old *w^1118^* flies received four strikes with different interinjury intervals ranging from 5 min to 48 hr. U indicates uninjured flies at the 24 hr time point. (A and B) Survival percentage of injured (colored lines) and uninjured (black line) flies. At least 200 flies were analyzed for each condition. Error bars represent the SEM. The dotted line indicates 24 hr after all flies received four strikes. (C and D) Mortality percentage at 24 hr after flies from all interinjury interval conditions received four strikes. The data were extracted from (A and B), respectively, at the time point indicated by the dotted vertical lines. Each data point is the average and SEM of at least three biological replicates. In (C), the 2, 8, and 24 hr time points were significantly increased compared with the 5 min time point (*P* < 0.05, one-tailed *t*-test). In (D), the 2 and 8 hr time points were significantly increased compared with the 5 min time point (*P* < 0.05, one-tailed *t*-test).

Our standard TBI protocol consists of four strikes from the HIT device separated by 5-min intervals (Katzenberger *et al.* 2013). Previously, we found that the MI_24_ for 0–7 d old *w^1118^* flies (a common laboratory strain) is not significantly altered by interinjury intervals of 5, 10, 20, 30, or 60 min. In contrast, as shown in Figure 1, increasing the interinjury interval to 2 hr significantly increased the mortality percentage at 24 hr after the final strike for flies injured at either 0–3 or 20–23 d old. Furthermore, the enhancing effect progressively diminished when the interval was increased to 8, 24, and 48 hr, returning to the 5-min level at 48 hr. These data indicate that secondary injury mechanisms have peak activity 1–8 hr after primary injuries.

**Figure 2:**
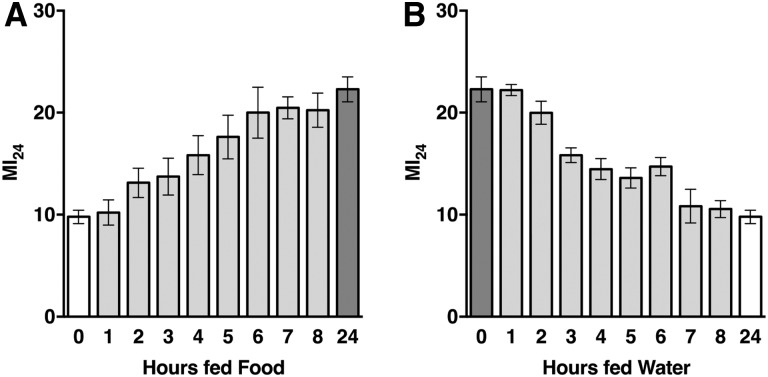
The duration of feeding water and food following primary injuries affects the MI_24_ of younger *w^1118^* flies. (A) Flies were fed food for the indicated amount of time following primary injuries and water for the remaining time up to 24 hr. (B) Flies were fed water for the indicated amount of time following primary injuries and food for the remaining time up to 24 hr. White bars represent 24 hr fed water, dark gray bars represent 24 hr fed food, and light gray bars represent a mixture of time fed water and food. Each data point is the average and SEM of at least three biological replicates. In (A), the 4, 5, 6, 7, 8, and 24 hr time points were significantly increased compared with the 0 hr time point (*P* < 0.05, one-tailed *t*-test). In (B), the 3, 4, 5, 6, 7, 8, and 24 hr time points were significantly decreased compared with the 0 hr time point (*P* < 0.05, one-tailed *t*-test).

As an independent means of determining the timing of secondary injuries that cause mortality, we changed the diet after primary injuries. Previously, we found that 0–7 d old *w^1118^* flies fed water following the standard TBI protocol had a significantly lower MI_24_ than equivalent flies fed molasses food, which consists primarily of molasses, yeast, cornmeal, and agar (Katzenberger *et al.* 2015a). To determine when diet affects the MI_24_ during the 24-hr period, we varied the amount of time 0–7 d old *w^1118^* flies were fed water or molasses food, hereafter referred to as “food.” Relative to flies fed water for 24 hr, flies fed food for the initial 2 hr and water for the subsequent 22 hr had a higher MI_24_ (Figure 2A). Increasing the amount of time flies were fed food by 1-hr increments further increased the MI_24_, with a plateau at 6 hr. The converse experiment showed similar timing. Relative to flies fed food for 24 hr, flies fed water for the initial 2 hr and food for the subsequent 22 hr had a lower MI_24_ (Figure 2B). Increasing the amount of time flies were fed water by 1-hr increments further decreased the MI_24_, with a plateau at 7 hr. These data indicate that secondary injury mechanisms have peak activity 1–7 hr after primary injuries.

### Age- and diet-regulated mechanisms promote mortality via different secondary injuries

**Figure 3:**
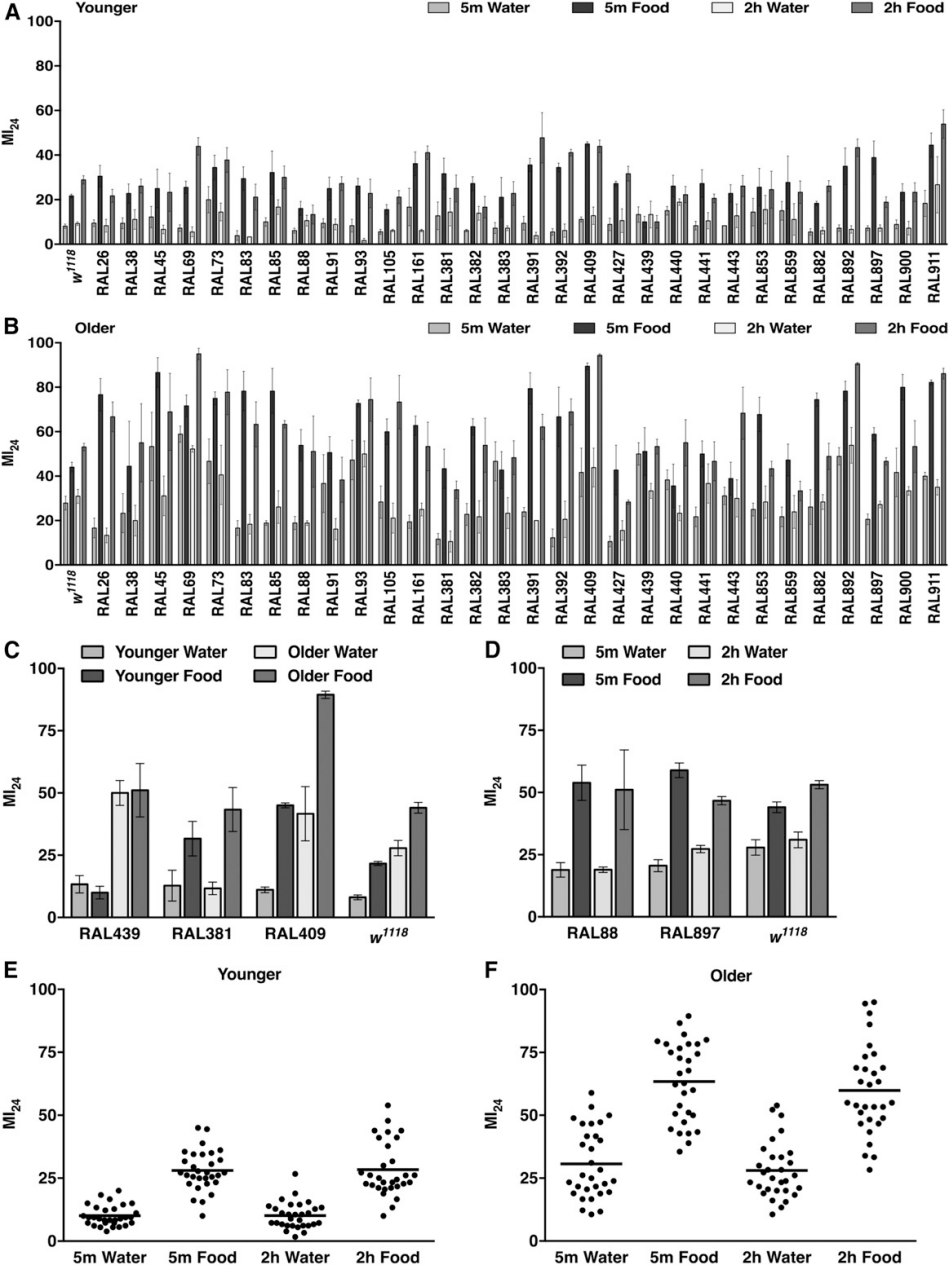
Age, diet, and interinjury interval have different effects on the MI_24_ in different genetic backgrounds. Shown is the MI_24_ of 30 RAL lines subjected to TBI with 5 min or 2 hr interinjury intervals at (A) younger or (B) older ages and subsequently fed water or food. Each data point is the average and SEM of at least three biological replicates. (C) Representative fly lines from (A and B) whose MI_24_ was affected by age but not diet (RAL439), diet but not age (RAL381), or both age and diet (RAL409 and *w^1118^*) following the standard injury protocol. (D) Representative fly lines from (B) whose MI_24_ was not affected by interinjury interval (RAL88), reduced at 2 hr *vs.* 5 min under the food condition (RAL897), or increased at 2 hr *vs.* 5 min under the food condition (*w^1118^*). The average MI_24_ for the set of 30 RAL lines treated at (E) younger or (F) older ages. Each dot represents a single RAL line and the horizontal line indicates the average among fly lines. The average MI_24_ was significantly different (*P* < 0.0001, one-tailed *t*-test) between younger water and younger food as well as between younger water and older water for both 5 min and 2 hr intervals. In contrast, the average MI_24_ was not significantly different (*P* > 0.05, one-tailed *t*-test) between 5 min and 2 hr intervals for both younger and older flies fed either water or food.

To assess the generality of the findings from *w^1118^* flies, we determined the effects of age, diet, and interinjury interval on the MI_24_ of 30 inbred RAL lines from the DGRP (Mackay *et al.* 2012). Figure 3 shows the data for individual lines as well as the average of all lines, and Table 1 shows the percentage of lines that had significantly different MI_24_s among age, diet, and interinjury interval conditions. Table 1 is based on *P*-values derived from Figure 3, A and B and shown in Table S1. Hereafter, we refer to 0–7 d old flies as “younger” flies and 20–27 d old flies as “older” flies.

For most RAL lines, older flies had a significantly higher MI_24_ than younger flies (Figure 3, A and B and Table 1). For every line, the MI_24_ either increased or did not change between younger and older flies. The average MI_24_ for all lines was significantly higher for older than for younger flies under the same diet and interinjury interval conditions (Figure 3, E and F). Thus, age-regulated mechanisms that enhance mortality-causing secondary injuries are active in most but not all genotypes.

Most RAL lines also had a significantly higher MI_24_ when fed food *vs.* water (Figure 3, A and B and Table 1). For every line, the MI_24_ either increased or did not change between water and food. The average MI_24_ for all lines was significantly higher for food than for water under the same age and interinjury interval conditions (Figure 3, E and F). Thus, diet-regulated mechanisms that enhance mortality-causing secondary injuries are active in most but not all genotypes. Taken together, these studies indicate that age and diet independently promote secondary injuries. With the standard TBI protocol, some lines (such as RAL439) only had an age-regulated increase in MI_24_ and others (such as RAL381) only had a diet-regulated increase in MI_24_, indicating that secondary injuries promoted by age and diet are controlled by independent genetic mechanisms (Figure 3C). Furthermore, some lines (such as RAL409 and *w^1118^*) had both age- and diet-regulated increases in MI_24_, indicating that secondary injuries promoted by age and diet are controlled by genetic mechanisms that are not mutually exclusive.

To determine whether secondary injuries promoted by age and diet function additively or synergistically, we analyzed the average MI_24_ data (Figure 3, E and F). For simplicity, younger flies fed water = younger water; younger flies fed food = younger food; older flies fed water = older water; and older flies fed food = older food. We added the average change in MI_24_ due to age (*i.e.*, the average MI_24_ for older water minus younger water) to the average change in MI_24_ due to diet (*i.e.*, the average MI_24_ for younger food minus younger water) and compared that to the average MI_24_ due to age and diet (*i.e.*, the average MI_24_ for older food minus younger water). For the 5-min interinjury interval, the individual effects of age and diet added together (20.6 + 18.0 = 38.6) were substantially less than the combined effect of age and diet (53.4). Similarly, for the 2-hr interinjury interval, the individual effects of age and diet added together (17.9 + 18.2 = 36.1) were substantially less than the combined effect of age and diet (49.7). Identical analyses of the 10 and six RAL lines that had both age- and diet-regulated increases in MI_24_ with 5-min and 2-hr interinjury intervals, respectively, also revealed more than additive effects of age and diet on the MI_24_. These data indicate that when age- and diet-regulated secondary injuries co-occur, they synergistically increase mortality.

Lastly, interinjury interval did not affect the MI_24_ for almost all lines, including RAL88 (Figure 3, A, B, and D and Table 1). The average MI_24_ for all lines was not significantly different between interinjury intervals of 5 min and 2 hr under the same age and diet conditions (Figure 3, E and F). In all cases where interinjury interval affected the MI_24_, flies were fed food, which suggests that secondary injuries affected by interinjury interval are dependent on food (Table 1). Interestingly, for older flies fed food, increasing the interinjury interval from 5 min to 2 hr reduced the MI_24_ for three lines (RAL853, RAL882, and RAL897), suggesting that in some genetic backgrounds, an initial primary injury can inhibit secondary injuries caused by a subsequent primary injury (Figure 3, A, B, and D and Table 1). Thus, in a small minority of genotypes, interinjury interval affects diet-regulated secondary injuries that cause mortality.

### Innate immune response genes dominate the early transcriptional response to primary injuries

**Figure 4:**
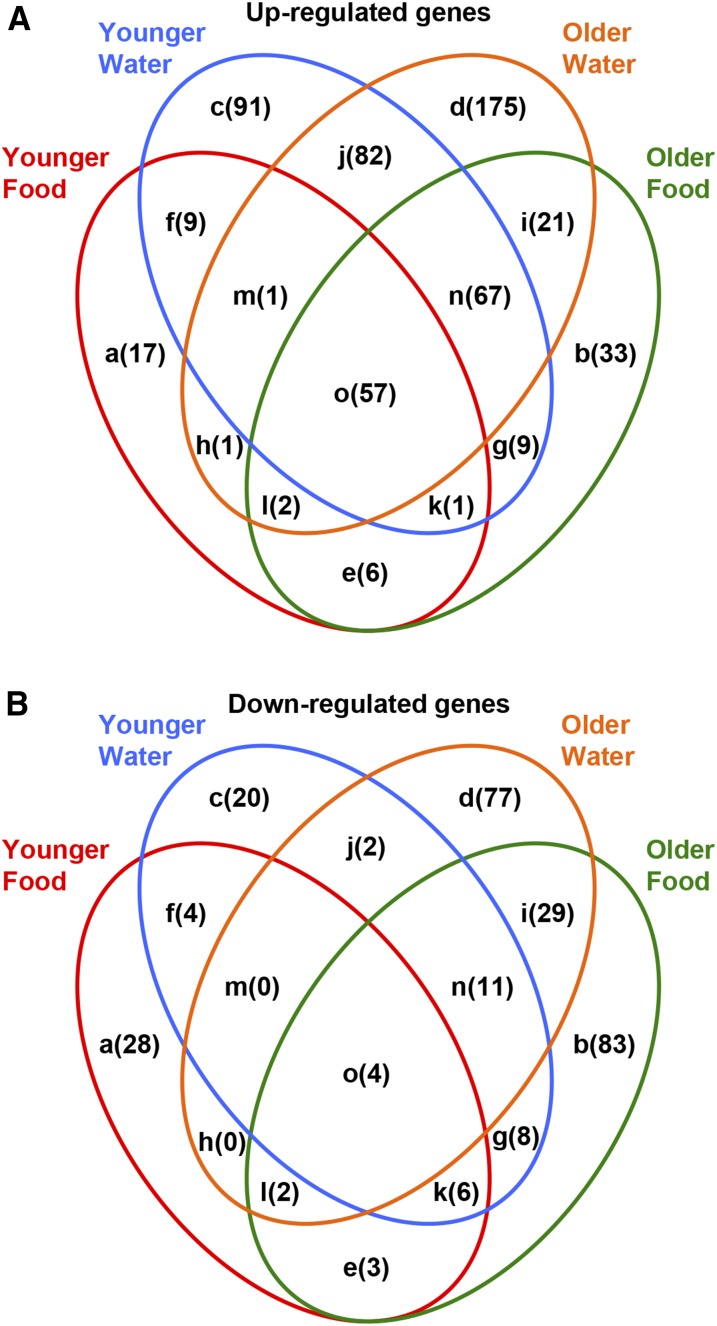
Overview of significant changes in gene expression after primary injuries in *w^1118^* flies, as determined by RNA-seq at 2 hr postinjury for flies fed water and 4 hr postinjury for flies fed food. Indicated in parentheses is the number of genes whose expression was (A) up-regulated or (B) down-regulated more than twofold, with an FDR *P* < 0.05, following primary injuries among the four indicated conditions. Lowercase letter designations correspond to Table S2 and Table S3, which list up-regulated and down-regulated genes, respectively. Table 2 contains the complete list of up-regulated and down-regulated genes that were common to all four conditions, and Table 3 contains a partial list of innate immune response genes that were common to a subset of conditions.

To gain insight into the mechanisms activated by primary injuries, we used high-throughput RNA-seq to identify gene expression changes induced by primary injuries. Genome-wide expression profiles of injured and uninjured *w^1118^* flies were compared under four conditions: (1) younger water, (2) younger food, (3) older water, and (4) older food. These conditions were examined because they produced different MI_24_s when primary injuries were held constant using the standard injury protocol (four strikes with 5-min interinjury intervals) (Figure 3C). Male flies were used to exclude gene expression differences between sexes, and mRNA from whole flies was used because the response to primary injuries is likely to involve protein-coding genes and not be limited to the brain (Owusu-Ansah and Perrimon 2015). Lastly, younger and older water conditions were examined 2 hr after primary injuries and younger and older food conditions were examined 4 hr after primary injuries. In retrospect, it would have been better to use the same time point under both conditions to be certain that the observed differences in gene expression are due exclusively to the feeding condition and not the time differential. With this potential caveat in mind, analyses of three independent biological replicates for each condition revealed that in at least one condition, 849 genes met the criteria of a more than twofold change in expression between injured and uninjured flies with a false discovery rate (FDR) *P*-value ≤ 0.05. A total of 572 genes were up-regulated and 277 genes were down-regulated. Figure 4 presents the number of genes that overlap among the four conditions, and Table S2 and Table S3 contain lists of up-regulated and down-regulated genes, respectively.

Almost all 57 genes that were up-regulated under all conditions are part of the innate immune response (Figure 4 and Table 2), including transcriptional targets of the Toll pathway [*e.g.*, AMPs, Immune-induced molecules (IMs), Bomanins (Boms), and Thioester-containing protein 2 (TEP2)], the Imd pathway (*e.g.*, AMPs), the JAK-STAT pathway (*e.g.*, TEP2), and the Mekk1 pathway [*e.g.*, CG13905, CG14957, CG15829, Stress-induced DNase (SID), and Urate oxidase (Uro)], as well as pattern recognition receptors involved in the recognition of DAMPs and pathogen-associated molecular patterns (PAMPs) in the Toll pathway [*e.g.*, Peptidoglycan recognition protein SA (PGRP-SA) and Gram-negative binding protein-like 3 (GNBP-like3)] and genes that regulate the JAK-STAT pathway [*e.g.*, Suppressor of cytokine signaling at 36E (Socs36E)] (De Gregorio *et al.* 2001, 2002; Agaisse and Perrimon 2004; Lemaitre and Hoffmann 2007; Bier and Guichard 2012; Stec *et al.* 2013; Chakrabarti *et al.* 2014; Kurata 2014; Clemmons *et al.* 2015). Also included were genes up-regulated by pathogen infection that control proteolysis, cell growth, and oxidative stress. In addition, two of the four genes that were down-regulated in all conditions are involved in the innate immune response: *Ser8*, which encodes a serine protease induced by bacterial infection; and *CG4950*, to which encodes a transcriptional target of the Mekk1 pathway following bacterial infection (De Gregorio *et al.* 2001; Chakrabarti *et al.* 2014). These data indicate that, regardless of age at the time of primary injuries or diet afterward, innate immune response pathways mediate the dominant, early transcriptional response to primary injuries.

**Table 2:** Genes affected in all conditions

	Younger*^a^*		Older	
	Water	Food	Water	Food
MI_24_	8.1 ± 0.9	21.7 ± 0.8	27.9 ± 3.1	44.0 ± 2.2
Antimicrobial peptides (AMPs)				
AttA	15.3	4.8	33.1	7.3
AttB	8.6	3.7	31.4	7.8
AttC	8.1	5.6	13.1	9.2
CecA2	10.4	2.9	37.8	9.9
CecB	33.6	2.6	72.3	4.8
CecC	11.7	5.4	100.7	6.7
Dpt	7.1	5.9	7.9	6.1
DptB	6.1	4.1	11.7	6.8
Dro	7.0	4.9	7.1	5.0
Drs	10.2	5.9	4.0	5.7
Mtk	6.1	6.1	4.1	5.2
Immune-induced molecules (IMs)				
IM1 (Bom)	7.3	4.2	2.8	4.4
IM2 (Bom)	4.5	2.7	2.6	3.2
IM3 (Bom)	4.1	2.4	2.7	3.1
IM4	4.9	3.1	2.3	3.3
IM10	2.5	3.5	3.2	3.1
IM14	5.9	3.9	3.3	4.2
IM18	2.3	2.3	4.7	3.9
IM23 (Bom)	9.4	5.0	3.5	5.6
CG10332	2.3	2.3	4.7	3.9
CG15065 (Bom)	5.2	3.4	2.4	3.3
CG16713	6.7	3.0	4.2	5.7
CG33470	5.1	3.2	2.8	3.4
CG43165	5.1	4.8	9.2	10.7
CG43202 (Bom)	7.3	3.9	6.0	4.5
Other peptides				
Edin	5.5	9.0	11.2	7.9
Listericin	4.7	2.1	2.5	2.5
CG13324	5.5	2.3	2.5	2.8
CG14957	12.4	2.4	3.8	4.7
CG15829	6.8	2.4	3.9	3.2
CG16978	8.3	2.2	4.7	5.1
CG43175	2.4	3.5	3.0	2.2
CG43236	6.3	3.8	5.4	4.5
Pathogen recognition receptors				
GNBP-like	7.6	4.0	8.0	5.4
PGRP-SA	7.5	3.1	3.7	3.1
Complement-like				
Tep2	3.5	2.2	2.2	2.4
Serine proteases				
Ser8	−3.2	−2.1	−4.7	−3.4
SP10	24.0	7.7	16.6	10.1
SPH93	6.4	3.0	3.0	5.2
CG18557	10.7	3.8	5.3	12.0
Serine protease inhibitor				
Spn88Eb	6.1	3.1	3.3	4.5
Growth control				
Ets21C	14.2	4.7	15.0	7.0
Gadd45	3.5	2.0	3.8	2.4
Oxidative stress				
GstD2	4.1	2.6	2.5	2.2
Other				
Fst	17.6	5.0	10.5	8.4
NimB1	2.7	2.3	2.0	2.1
NimC2	−2.3	−2.5	−2.3	−3.8
SID	5.9	2.9	5.9	6.0
Socs36E	4.6	2.1	4.4	3.0
Uro	6.1	3.1	3.0	2.7
CG4950	−2.7	−2.3	−3.3	−5.1
CG5550	6.3	2.2	8.3	2.5
CG10182	8.5	2.6	31.7	5.4
CG13641	2.2	2.1	3.3	2.1
CG13905	5.0	2.6	2.3	2.6
CG15263	−2.7	−2.3	−4.8	−2.6
CG16772	5.0	2.4	5.0	3.1
CG18067	4.3	3.2	2.3	2.5
CG30026	4.9	2.6	2.9	3.3
CG34054	5.4	2.5	3.8	3.9
CG43085	5.2	9.2	3.8	3.5

a Fold change in expression between injured and uninjured flies.

### Age and diet affect expression of innate immune response genes following primary injuries

Genes involved in secondary injury pathways that cause mortality were predicted to have different fold changes in expression between younger and older flies or between water and food diets, conditions that had different MI_24_s. Differentially expressed genes included most of the 57 genes that had increased expression in all conditions (Table 2). In fact, 88% (50 of 57) of these genes had a larger fold change in younger water compared with younger food conditions, indicating that fasting (*i.e.*, the water condition) enhances and ingestion of nutrients (*i.e.*, the food condition) suppresses activation of the innate immune response by primary injuries. In addition, 60% (34 of 57) of these genes, but only 18% (two of 11) of AMP genes had a greater fold change in younger water compared with older water conditions, indicating that aging reduces the ability of fasting to enhance activation of the innate immune response by primary injuries.

Differentially expressed genes also included other innate immune response genes that changed expression in a subset of conditions (Table 3). Following primary injuries, all six genes that were up-regulated by food but not by water are involved in the innate immune response (Table 3). Four of the six genes, Turandot A (TotA), TotC, TotM, and TotX, are part of the eight-member Tot family of secreted peptides that are induced by a variety of stresses, including bacterial infection, and are activated by the Imd, JAK-STAT, and Mekk1 pathways (Ekengren and Hultmark 2001; Brun *et al.* 2006; Chakrabarti *et al.* 2016). The other two genes are Diedel, which is a cytokine that represses the Imd pathway in response to virus infection, and CG11459, which is a predicted Cathepsin-like peptidase induced by bacterial infection (De Gregorio *et al.* 2001; Lamiable *et al.* 2016). Genes that were up-regulated by water but not by food include both positive [*e.g.*, Spätzle (Spz) and PGRP-SD] and negative [*e.g.*, Cactus (Cact) and Necrotic (Nec)] regulators of the Toll pathway, as well as p38c, a MAP kinase in the Mekk1 pathway that activates the production of ROS (Lemaitre and Hoffmann 2007; Chakrabarti *et al.* 2014). Genes that were down-regulated in older but not in younger flies include Eater, Nimrod C1 (NimC1), and Scavenger receptor class C, type 1 (Sr-C1), which are three of the eight receptors found on the surface of macrophages that are involved in binding and eliminating pathogens by phagocytosis, as well as Lectin-24Db and Lectin-33A, which are secreted C-type Lectins that function as pattern recognition receptors to mediate pathogen encapsulation by hemocytes (Ao *et al.* 2007; Ferrandon *et al.* 2007). Genes that were up-regulated in older but not in younger flies include PGRP-LB, which inhibits activation of the Imd pathway in response to bacterial infection by cleaving DAP-type peptidoglycans, and the cytokine Unpaired 2 (Upd2), which is a transcriptional target of the Jun kinase (JNK) pathway and activates the JAK-STAT pathway (Zaidman-Rémy *et al.* 2006; Rajan and Perrimon 2012). Lastly, in other subsets of conditions, up-regulated genes included other AMPs, Tots, and Upds, as well as Relish (Rel), which is the NF-κB transcription factor in the Imd pathway (Ferrandon *et al.* 2007; Lemaitre and Hoffmann 2007). These data indicate that age- and diet-regulated mechanisms modulate the transcriptional output of the Toll, Imd, JAK-STAT, JNK, and Mekk1 pathways following primary injuries. Furthermore, regulation of innate immune response pathways following primary injuries is likely to be complex, since gene expression changes predict both positive and negative regulation of the pathways.

**Table 3:** Genes affected in a subset of conditions

	Younger*^a^*		Older	
	Water	Food	Water	Food
MI_24_	8.1 ± 0.9	21.7 ± 0.8	27.9 ± 3.1	44.0 ± 2.2
Water-specific genes				
Cact	2.5		2.5	
Nec	2.9		2.0	
PGRP-SD	2.1		2.2	
Spz	2.7		2.4	
Food-specific genes				
Diedel		154.0		8.1
TotA		3.7		3.3
TotC		3.9		2.2
TotM		13.8		5.0
TotX		2.3		3.0
CG11459		2.7		2.7
Older-specific genes				
Eater			−2.5	−2.1
Lectin-24Db			−2.6	−2.2
Lectin-33A			−2.1	−3.0
NimC1			−2.9	−2.1
PGRP-LB			2.7	2.2
Sr-Cl			−4.9	−3.0
Upd2			10.9	5.1

a Fold change in expression between injured and uninjured flies.

### Primary injuries induce rapid biphasic activation of innate immune response genes

**Figure 5:**
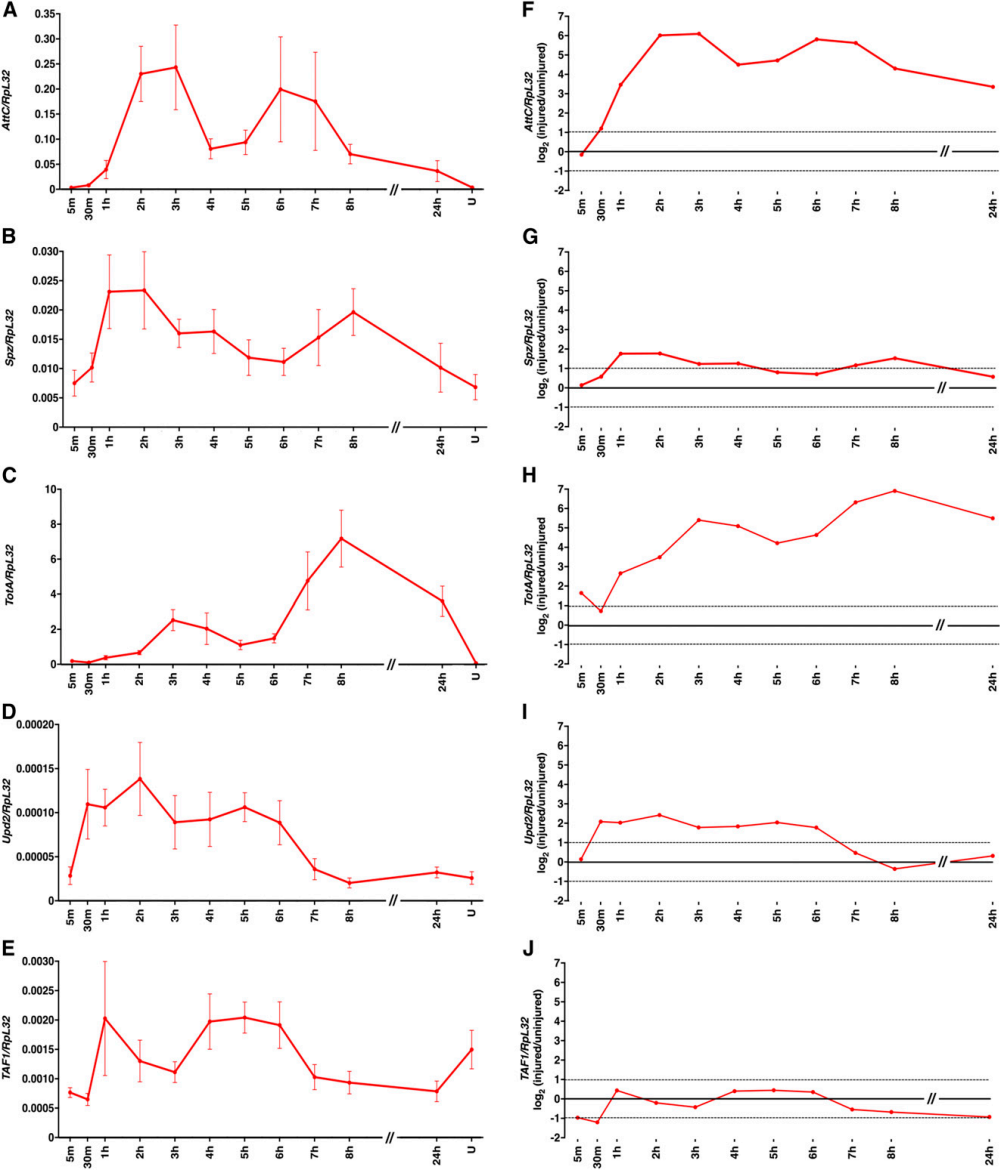
Innate immune response genes had different expression profiles over the 24 hr period following primary injuries in younger *w^1118^* flies fed food. (A–E) Expression of the indicated genes relative to RpL32 at the indicated time points following the standard injury protocol. Note that *y*-axis scales differ among graphs and that the *x*-axis is not to scale between 8 and 24 hr. U indicates uninjured flies at the 24 hr time point. Each data point is the average and SEM of at least three biological replicates. These data are shown in comparison to other conditions in Figure 6, Figure 7, Figure 8, Figure 9, and Figure 10. Note that the *y*-axis scales are different in Figure 6, Figure 7, Figure 8, Figure 9, and Figure 10. (F–J) Log_2_ expression difference in injured relative to uninjured flies of the indicated genes normalized to RpL32. Dotted lines indicate a twofold change in expression. Note that the *x*-axis is not to scale between 8 and 24 hr.

We used RT-qPCR to more thoroughly examine gene expression following primary injuries. These experiments are important because fold changes in expression between uninjured and injured flies at one time point after primary injuries may not be the same as at other time points, and also may not be as relevant to fly physiology as absolute levels of expression. We focused on genes that encode secreted proteins in the innate immune response because excessive secretion of proinflammatory cytokines in mammals is believed to cause multiple organ dysfunction syndrome and mortality following TBI (Lu *et al.* 2009). Younger flies were subjected to the standard TBI protocol, fed food, and analyzed by RT-qPCR at times encompassing the 1–8 hr peak period of secondary injuries. A mixture of male and female flies was used, rather than just males, as was used for RNA-seq, because a large number of flies was needed for the analysis and male and female flies have the same MI_24_ (Katzenberger *et al.* 2013). To determine absolute levels of expression, mRNA levels of each gene were normalized to those of Ribosomal protein L32 (RpL32) (Figure 5, A–E). To establish the level of variation inherent in the assay, we examined a generally expressed gene TBP-associated factor 1 (TAF1) (Papai *et al.* 2011). TAF1 expression changed less than twofold between uninjured and injured flies at almost all points in the time course, whereas the AMP Attacin C (AttC), the Toll ligand Spz, the secreted peptide TotA, and the cytokine Upd2 changed expression more than twofold at most points in the time course (Figure 5, F–J). These data confirm the RNA-seq finding that primary injuries significantly increase expression of innate immune response genes during the 1–8 hr peak period of secondary injuries that cause mortality.

Expression of AttC, Spz, TotA, and Upd2 was rapidly up-regulated with distinct profiles following primary injuries under the younger food condition (Figure 5, A–E). Substantial up-regulation occurred by 30 min for Upd2 and by 1 hr for AttC, Spz, and TotA (Figure 5, F–I). Expression of AttC, Spz, and TotA was biphasic, with peaks at 1–4 and 6–8 hr. In contrast, Upd2 had a single extended peak at 30 min–6 hr. The later peaks of AttC and Spz were similar in magnitude to the earlier peaks, but the later peak of TotA was considerably higher than the earlier peak. Finally, expression of AttC and TotA, but not Spz and Upd2, remained up-regulated at 24 hr. These data lead to several conclusions: (1) biphasic expression patterns, as observed in mammals (Hu *et al.* 2014), indicate temporally distinct modes of PAMP, DAMP, or ROS production following primary injuries; (2) diverse expression patterns indicate complex regulation of the Toll, Imd, JAK-STAT, JNK, and Mekk1 pathways following primary injuries; and (3) up-regulated expression at 24 hr indicates that PAMPs, DAMPs, or ROS continue to be produced in flies that survive following primary injuries.

### Expression of specific innate immune response genes may underlie age- and diet-regulated mortality-associated secondary injuries

**Figure 6:**
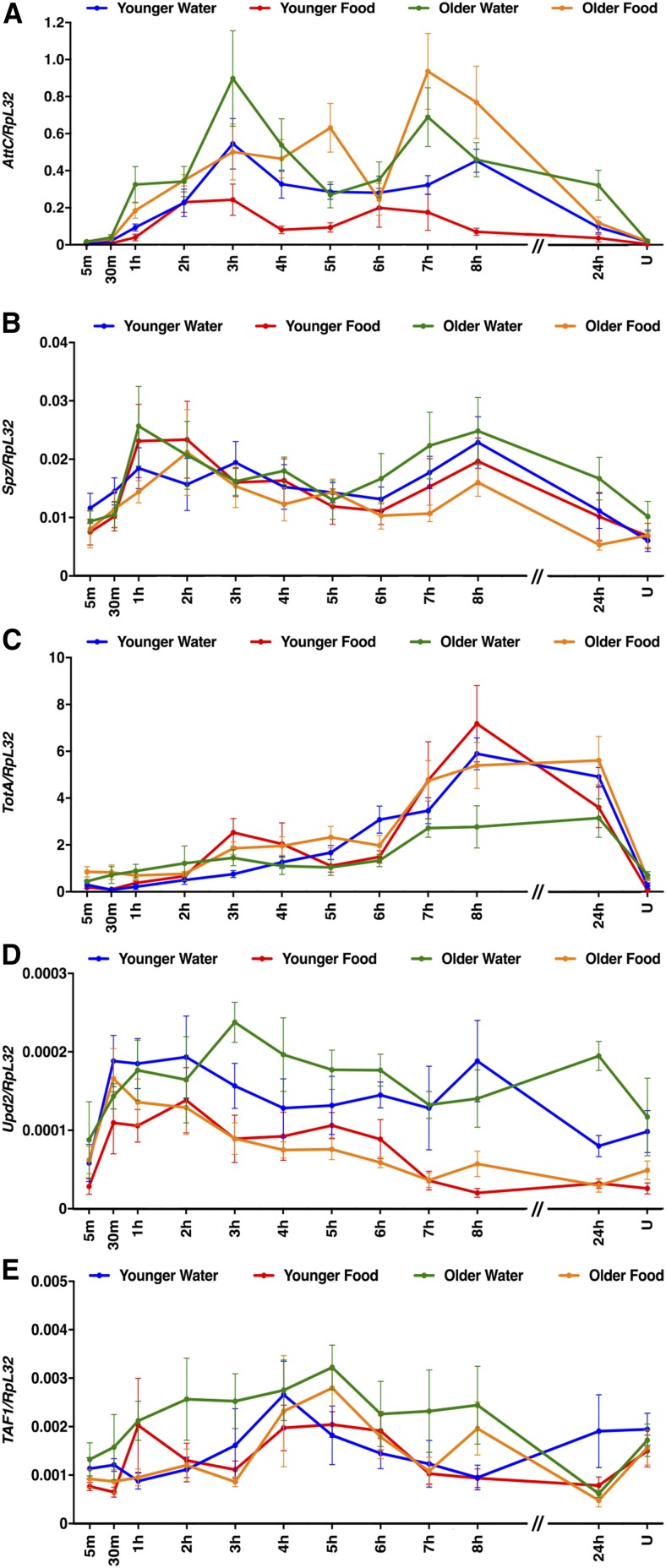
Age and diet affected expression of some innate immune response genes following primary injuries. (A–E) Expression of the indicated genes relative to RpL32 following the standard injury protocol in *w^1118^* flies under the indicated conditions. Note that *y*-axis scales are different among graphs and that the *x*-axis is not to scale between 8 and 24 hr. Each data point is the average and SEM of at least three biological replicates. RNA samples used for this analysis were also used in Figure 7, Figure 8, Figure 9, and Figure 10.

We used RT-qPCR to analyze the 24-hr time course of expression of AttC, Spz, TotA, Upd2, and TAF1 after the standard injury protocol in younger water, younger food, older water, and older food conditions (Figure 6). Genes were considered to have changed expression in a primary injury-dependent manner if their average expression at 1–8 hr was more than twofold different than their average expression at 5–30 m and at the 24 hr time point in uninjured flies (Table S4). By these criteria, expression of Spz, Upd2, and TAF1 was not affected by primary injuries under any conditions, and expression of AttC and TotA was affected by primary injuries under all conditions. The average expression of AttC at 1–8 hr was lower in younger water than older water conditions and higher in younger water than younger food conditions. Paradoxically, these data indicate that if age- and diet-regulated changes in expression of AttC affect mortality, AttC would promote mortality in an age-regulated manner and inhibit mortality in a diet-regulated manner. An analogous but opposite paradox occurred with TotA, whose expression was higher for younger water than older water conditions but lower for younger water than younger food conditions. Thus, these data demonstrate that expression of AttC and TotA is altered by age- and diet-regulated mechanisms, but the paradoxical relationship between expression and the MI_24_ suggests that altered expression of these genes individually does not correlate with secondary injuries that cause mortality. This outcome is not surprising, given the complexity of changes in gene expression following primary injuries. Instead, mortality caused by secondary injuries is most likely the outcome of a complicated pattern of gene expression involving a large suite of genes.

**Figure 7:**
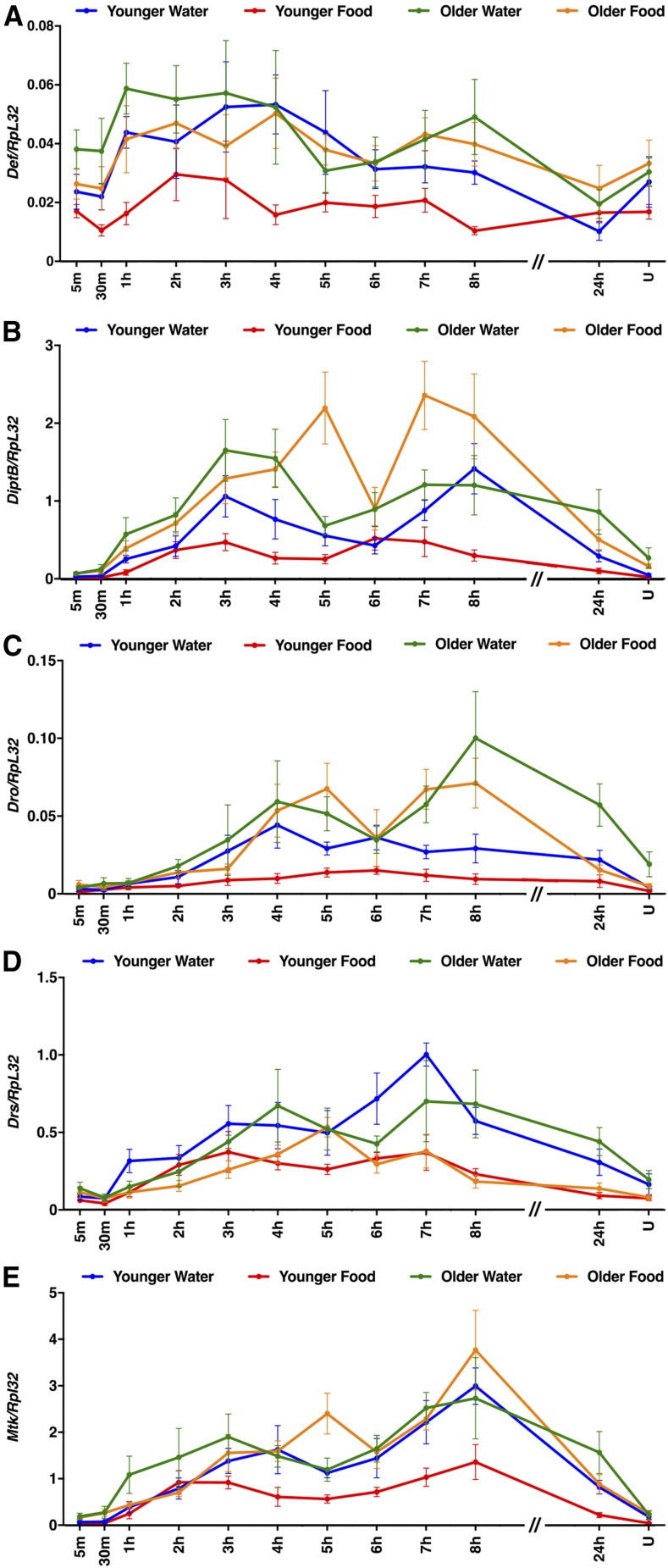
Age and diet affected expression of some AMP family genes following primary injuries. (A–E) Expression of the indicated genes relative to RpL32 following the standard injury protocol in *w^1118^* flies under the indicated conditions. Note that *y*-axis scales are different among graphs and that the *x*-axis is not to scale between 8 and 24 hr. U indicates uninjured flies at the 24 hr time point. Each data point is the average and SEM of at least three biological replicates.

**Figure 8:**
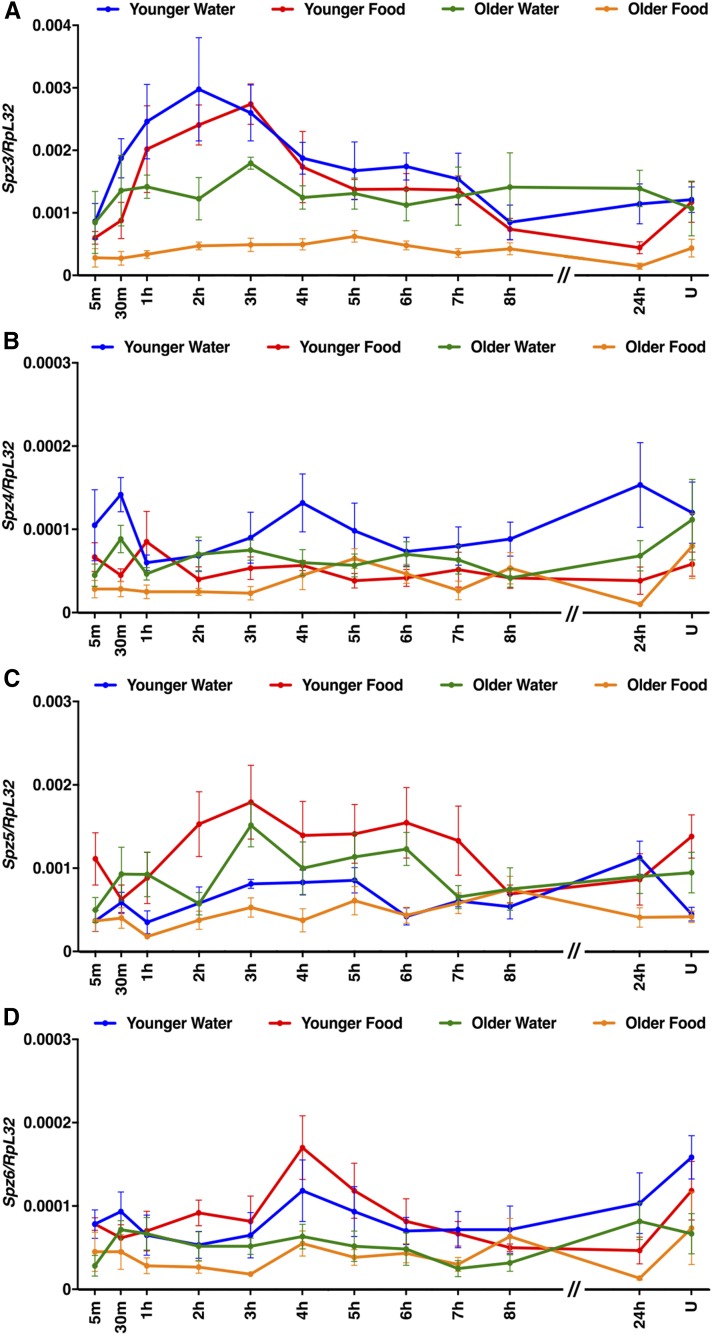
Age and diet affected expression of some Spz family genes following primary injuries. (A–D) Expression of the indicated genes relative to RpL32 following the standard injury protocol in *w^1118^* flies under the indicated conditions. Note that *y*-axis scales are different among graphs and that the *x*-axis is not to scale between 8 and 24 hr. U indicates uninjured flies at the 24 hr time point. Each data point is the average and SEM of at least three biological replicates.

**Figure 9:**
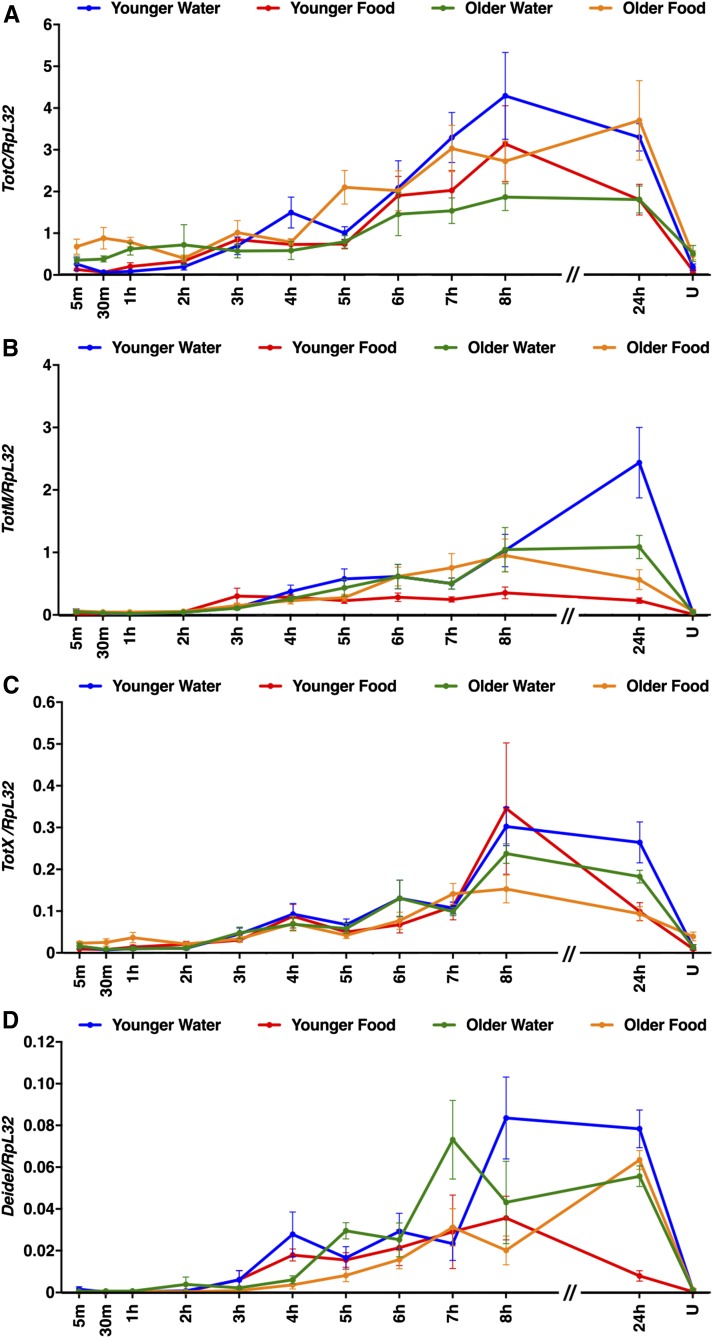
Age and diet affected expression of Tot family genes and Diedel following primary injuries. (A–D) Expression of the indicated genes relative to RpL32 following the standard injury protocol in *w^1118^* flies under the indicated conditions. Note that *y*-axis scales are different among graphs and that the *x*-axis is not to scale between 8 and 24 hr. U indicates uninjured flies at the 24 hr time point. Each data point is the average and SEM of at least three biological replicates.

**Figure 10:**
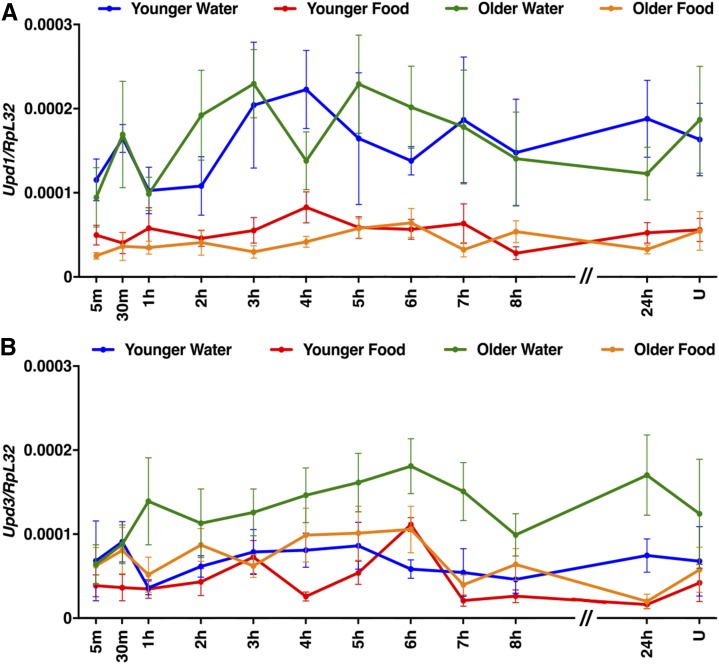
Age and diet did not affect expression of Upd family genes following primary injuries. (A and B) Expression of the indicated genes relative to RpL32 following the standard injury protocol in *w^1118^* flies under the indicated conditions. Note that the *x*-axis is not to scale between 8 and 24 hr. U indicates uninjured flies at the 24 hr time point. Each data point is the average and SEM of at least three biological replicates.

To assess the generality of the findings with AttC, Spz, TotA, and Upd2, we examined expression of other members of the AMP, Spz, Tot, and Upd families, as well as Diedel (Figure 7, Figure 8, Figure 9, and Figure 10). Among these genes, primary injury-induced expression was not affected under any condition for Defensin (Def) (Figure 7A); Spz3, Spz4, Spz5, and Spz6 (Figure 8); and Upd1 and Upd3 (Figure 10), and expression was only affected under a subset of conditions for TotC and TotX (Figure 9, A and C). In contrast, primary injury-induced expression was affected under all condition for Diptericin B (DiptB), Drosocin (Dro), Drosomycin (Drs), and Metchnikowin (Mtk) (Figure 7, B–E); TotM (Figure 9B); and Diedel (Figure 9D). Like AttC, these data paradoxically indicate that DiptB, Dro, and Mtk both inhibit and promote mortality. On the other hand, these data indicate that Drs, TotM, and Diedel only inhibit mortality. Expression of Drs, TotM, and Diedel decreased with age and diet, while mortality increased with age and diet. Thus, a subset of innate immune response genes might inhibit secondary injuries that cause mortality.

## Discussion

We used a fly model to investigate why TBI causes acute mortality. In humans and flies, the probability of mortality following TBI is associated with age and blood/hemolymph glucose level, which is influenced by diet (Susman *et al.* 2002; Hukkelhoven *et al.* 2003; Griesdale *et al.* 2009; Dhandapani *et al.* 2012; Katzenberger *et al.* 2013, 2015a; Wang *et al.* 2013; Borsage *et al.* 2015; Chong *et al.* 2015). Furthermore, studies of repetitive primary injuries in mammals and flies indicate that the time between primary injuries can affect the probability of mortality (Kanayama *et al.* 1996; Friess *et al.* 2009; Meehan *et al.* 2012; Huang *et al.* 2013; Weil *et al.* 2014; Bolton Hall *et al.* 2016) (Figure 1 and Figure 3). Thus, evolutionarily conserved age-, diet-, and interinjury interval-regulated mechanisms appear to promote secondary injuries that cause mortality. Our data address the timing of secondary injuries that cause mortality (Figure 1 and Figure 2), the genetic control of age-, diet-, and interinjury interval-regulated secondary injuries that cause mortality (Figure 3, Table 1, and Table S1), and gene expression changes associated with age- and diet-regulated secondary injuries that cause mortality (Figure 4, Figure 5, Figure 6, Figure 7, Figure 8, Figure 9, Figure 10, Table 2, Table 3, Table S2, and Table S3).

### Secondary injuries that cause mortality do not immediately follow primary injuries and are transient

Our data suggest that there is a lag period between primary and secondary injuries. The earliest change in expression of innate immune response genes after primary injuries occurred at 30 min for Upd2 (Figure 6, Figure 7, Figure 8, Figure 9, and Figure 10). Similarly, interinjury intervals of up to 1 hr did not affect the MI_24_ (Katzenberger *et al.* 2013), and changing the postprimary injury diet from water to food (or vice versa) for 1 hr did not affect the MI_24_ (Figure 2). Finally, we previously found that >98% of flies incapacitated by a single strike from the HIT device recovered mobility within 5 min, indicating that secondary injuries that cause mortality do not immediately follow primary injuries (Katzenberger *et al.* 2015a). Our data also suggest that secondary injuries are short-lived. Effects of interinjury interval on mortality were resolved 24–48 hr after primary injuries (Figure 1, C and D), effects of diet on mortality plateaued 5–7 hr after primary injuries (Figure 2), and expression of some innate immune response genes returned to basal levels 8 hr after primary injuries (Figure 6, Figure 7, Figure 8, Figure 9, and Figure 10). Moreover, mortality declined dramatically 24 hr after primary injuries (Figure 1, A and B). Previously, we found that the increase in glucose level in the hemolymph following primary injuries has similar kinetics, with a lag phase of 1–2 hr, a peak at 4–7 hr, and a return to basal levels at 8–16 hr (Katzenberger *et al.* 2015a). These data provide a temporal framework to identify biological processes underlying secondary injuries that cause acute mortality in flies.

### Genotype and diet affect the functional relationship between interinjury interval and mortality

Studies of TBI in mammals have shown that repetitive primary injuries produce worse outcomes than single injuries, and that increasing recovery time between repetitive injuries improves outcomes (Kanayama *et al.* 1996; Friess *et al.* 2009; Meehan *et al.* 2012; Huang *et al.* 2013; Weil *et al.* 2014; Bolton Hall *et al.* 2016). Our studies in flies support and extend these findings. First, we found that mortality following primary injuries occurred least frequently with either a short interinjury interval (*i.e.*, 5 min–1 hr) or a long interinjury interval (*i.e.*, 24–48 hr) (Figure 1) (Katzenberger *et al.* 2013). Lower mortality with a short interinjury interval has not been described for mammalian TBI models, possibly because sufficiently short interinjury intervals have not been tested; however, other outcomes are less severe with short interinjury intervals. For example, Huang *et al.* (2013) showed in a rat TBI model that hemorrhagic lesion volume and other outcomes are less severe with interinjury intervals of 1 or 7 d compared with 3 d. Second, we found that changing the interinjury interval did not affect mortality for most fly lines; 81% (25 of 31) of fly lines had the same MI_24_ with interinjury intervals of 5 min compared with 2 hr, regardless of age and diet conditions (Figure 3 and Table 1). In contrast, all studies of interinjury interval in mammals report an effect on outcomes, possibly because mortality and multiple strains have not been extensively tested. Third, we found that some fly lines had a lower MI_24_ with an interinjury interval of 2 hr compared with 5 min, suggesting that ongoing secondary injuries provide a conditioning effect; that is, secondary injuries condition the brain such that a subsequent injury has a reduced effect. A potentially related conditioning effect has been reported in a rat TBI model. Allen *et al.* (2000) showed that motor deficits are less severe in rats that receive a severe injury after a mild injury compared with only a severe injury. Lastly, we found that the interinjury interval affected the MI_24_ when flies were fed food but not water, indicating that food ingested after primary injuries is required for interinjury interval to have an effect on the MI_24_ (Table 1). Once again, analogous experiments have not yet been performed in mammals. Given the potential importance of the relationship between interinjury interval and outcomes in sports where athletes can sustain multiple primary injuries in a short period of time, these data in flies suggest that effects of genotype and diet should be explored in mammalian studies of interinjury interval (Bailes *et al.* 2014).

### Genetically distinct but functionally related mechanisms underlie age- and diet-regulated secondary injuries that cause mortality

We found that age at the time of primary injuries and diet afterward affected the MI_24_ to different extents in different fly lines (Figure 3 and Table 1). The MI_24_ of some fly lines was not affected by either age or diet, whereas the MI_24_ of other fly lines was affected many-fold by both age and diet. These data indicate that genetic variation is a major contributing factor to age- and diet-regulated secondary injuries that cause mortality, and that age- and diet-regulated mechanisms are genetically separable, although they may involve some of the same components. In support of these conclusions, sequence variation in several genes, including the cytokine IL-6, is associated with mortality in severe TBI patients, and sequence variation in the gene encoding Brain-derived neurotrophic factor interacts with age to influence mortality (Dardiotis *et al.* 2010; Dalla Libera *et al.* 2011; Garringer *et al.* 2013; Sperry *et al.* 2014; Failla *et al.* 2015, 2016). Links between genetic variation, diet, and mortality following primary injuries have not yet been reported in humans. However, diet has been linked to mortality. Meta-analysis of the timing of nutritional support shows that early nutrition following primary injuries reduces mortality compared with delayed nutrition (Wang *et al.* 2013). This finding contradicts the negative effect of early nutrition on mortality in flies (Figure 2), but the contradiction may be explained by different definitions of early nutrition, which was immediately after primary injuries in flies but often hours later in studies included in the meta-analysis. Thus, genetic control of aging processes and the response to diet appear to be a key determinants of heterogeneity in TBI outcomes.

We also found that age- and diet-regulated mechanisms function synergistically to promote secondary injuries that cause mortality (Figure 3 and Table 1). This finding suggests that mortality is determined by metabolic processes whose activity changes with age. A candidate metabolic process is ketosis, in which energy is provided by ketones rather than glucose (Prins and Matsumoto 2014). Fasting, which upregulates ketosis, is neuroprotective in a rat TBI model (Davis *et al.* 2008). Furthermore, Prins *et al.* (2005) found that rats fed a ketogenic diet immediately after primary injuries have reduced contusion volume relative to rats fed a standard diet, but the protective effect of the ketogenic diet only occurs in younger but not older rats. In the fly TBI model, fasting (*i.e.*, the water condition) may inhibit mortality by initiating use of ketones over glucose as an energy source. The inhibitory effect of fasting may be reduced in older compared with younger flies because of reduced capacity of older flies to convert to a ketone-metabolizing state, as is seen in a rat TBI model (Deng-Bryant *et al.* 2011). Moving forward, the genetic capabilities of flies make it possible to identify genes required for age- and diet-regulated mechanisms, as well as to determine the role of ketosis in secondary injuries that cause mortality.

### Expression of specific innate immune response genes may inhibit secondary injuries that cause mortality

We identified genome-wide changes in gene expression caused by mechanical injuries to adult flies from the HIT device. These data serve as a starting point for deciphering the cellular and molecular events triggered by mechanical injuries. The relevance of identified changes in gene expression to TBI is open to discussion because primary injuries from the HIT device are probably not limited to the brain. Thus, it is not possible to conclusively attribute changes in genes expression and mortality to primary injuries to the brain. Furthermore, because the gene expression studies were performed on whole flies, it is not known what cells and tissues are responsible for the changes in gene expression. Nevertheless, the observed effects on gene expression and mortality are consistent with brain injuries, as indicated by the documented phenotypic similarities between HIT device-injured flies and brain-injured rodents and humans. Furthermore, we previously reported that injuries from the HIT device activate expression of AMP genes in fly heads, and that injuries exclusively to the fly head are sufficient to cause mortality (Katzenberger *et al.* 2013, 2015a).

As has been shown in other systems, including rodent TBI models, we found that mechanical injuries elicited complex activation of the innate immune response in flies (Natale *et al.* 2003; Redell *et al.* 2013; White *et al.* 2013; Wong *et al.* 2016). In flies, complex activation of the innate immune response is demonstrated by the large number of genes that changed expression; expression of gene targets of multiple pathways, including the Toll, Imd, JAK-STAT, JNK, and Mekk1 pathways; expression of both positive and negative regulators of pathways; and different magnitudes and temporal patterns of gene expression (Figure 4, Figure 5, Figure 6, Figure 7, Figure 8, Figure 9, and Figure 10). Our data suggest that hidden within this complexity are a subset of genes that are relevant to mortality. For example, AMP genes fell into three classes: Def did not change expression under any condition; AttC, DiptB, Dro, and Mtk changed expression under all conditions and had increased expression with age and decreased expression with diet; and Drs changed expression under all conditions and had decreased expression with both age and diet (Figure 6 and Table 2). Thus, Drs was unique among AMP genes in having age- and diet-regulated expression that negatively correlated with the MI_24_. The lack of correlation between expression of AttC, DiptB, and Mtk and the MI_24_ is consistent with our previous finding that reduced expression of these genes in bacteria-free flies does not affect the MI_24_ (Katzenberger *et al.* 2015a). These data raise important questions: by what mechanisms do age and diet control transcription of Drs differently than other AMP genes and how might Drs function differently than other AMPs to inhibit mortality? The transcription mechanism is likely to involve the Toll pathway because Drs is predominantly regulated by the Toll pathway, as are secreted peptide-encoding genes in the Bom family that, like Drs, had age- and diet-regulated expression that negatively correlated with the MI_24_ (Lemaitre and Hoffmann 2007; Clemmons *et al.* 2015) (Table 2). The Drs-specific function may be related its ability to inactivate a voltage-gated sodium channel, since blocking upregulation of a sodium channel improves outcomes in a rat TBI model (Cohen *et al.* 2009; Huang *et al.* 2014).

Here, we have focused on innate immune response genes, but the RNA-seq experiments uncovered many other genes that could play important roles in secondary injuries that cause mortality. Included were genes involved in energy metabolism, oxidative stress, cell-cycle regulation, and protein homeostasis. Future genetic and molecular studies are needed to determine whether any of these changes in expression are necessary or sufficient to cause acute or chronic TBI outcomes.

## Supplementary Material

Supplemental Material

## References

[bib1] Agaisse H. Perrimon N., 2004 The roles of JAK/STAT signaling in *Drosophila* immune responses. Immunol. Rev. 198: 72–82. 15199955 10.1111/j.0105-2896.2004.0133.x

[bib2] Allen G. V. Gerami D. Esser M. J., 2000 Conditioning effects of repetitive mild neurotrauma on motor function in an animal model of focal brain injury. Neuroscience 99: 93–105. 10924955 10.1016/s0306-4522(00)00185-8

[bib3] Alluri H. Wiggins-Dohlvik K. Davis M. L. Huang J. H. Tharakan B., 2015 Blood-brain barrier dysfunction following traumatic brain injury. Metab. Brain Dis. 30: 1093–1104. 25624154 10.1007/s11011-015-9651-7

[bib4] Ao J. Ling E. Yu X. Q., 2007 *Drosophila* C-type lectins enhance cellular encapsulation. Mol. Immunol. 44: 2541–2548. 17287021 10.1016/j.molimm.2006.12.024 PMC1876673

[bib5] Bailes J. E. Dashnaw M. L. Petraglia A. L. Turner R. C., 2014 Cumulative effects of repetitive traumatic brain injury. Prog. Neurol. Surg. 28: 50–62. 24923392 10.1159/000358765

[bib6] Bansal V. Constantini T. Kroll L. Peterson C. Loomis W., 2009 Traumatic brain injury and intestinal dysfunction: uncovering the neuro-enteric axis. J. Neurotrauma 26: 1353–1359. 19344293 10.1089/neu.2008.0858 PMC2989839

[bib7] Bier E. Guichard A., 2012 Deconstructing host-pathogen interactions in *Drosophila*. Dis. Model. Mech. 5: 48–61. 21979942 10.1242/dmm.000406 PMC3255543

[bib8] Bolton Hall A. N. Joseph B. Brelsfoard J. M. Saatman K. E., 2016 Repeated closed head injury in mice results in sustained motor and memory deficits and chronic cellular changes. PLoS One 11: e0159442. 27427961 10.1371/journal.pone.0159442 PMC4948770

[bib9] Bosarge P. L. Shoultz T. H. Griffin R. L. Kerby J. D., 2015 Stress-induced hyperglycemia is associated with higher mortality in severe traumatic brain injury. J. Trauma Acute Care Surg. 79: 289–294. 26218699 10.1097/TA.0000000000000716

[bib10] Brooks J. C. Stauss D. J. Shavelle R. M. Paculdo D. R. Hammond F. M., 2013 Long-term disability and survival in traumatic brain injury: results from the National Institute on Disability and Rehabilitation Research model systems. Arch. Phys. Med. Rehabil. 94: 2203–2209. 23872079 10.1016/j.apmr.2013.07.005

[bib11] Brun S. Vidal S. Spellman P. Takahashi K. Tricoire H., 2006 The MAPKKK Mekk1 regulates the expression of Turandot stress genes in response to septic injury in *Drosophila*. Genes Cells 11: 397–407. 16611243 10.1111/j.1365-2443.2006.00953.x

[bib12] Chakrabarti S. Poidevin M. Lemaitre B., 2014 The *Drosophila* MAPK p38c regulates oxidative stress and lipid homeostasis in the intestine. PLoS Genet. 10: e1004659. 25254641 10.1371/journal.pgen.1004659 PMC4177744

[bib13] Chakrabarti S. Dudzic J. P. Li X. Collas E. J. Boquete J.-P., 2016 Remote control of intestinal stem cell activity by haemocytes in *Drosophila*. PLoS Genet. 12: e1006089. 27231872 10.1371/journal.pgen.1006089 PMC4883764

[bib14] Chong S. L. Harjanto S. Testoni D. Ng Z. M. Low C. Y., 2015 Early hyperglycemia in pediatric traumatic brain injury predicts for mortality, prolonged duration of mechanical ventilation, and intensive care stay. Int. J. Endocrinol. 215: 719476. 10.1155/2015/719476 PMC4446478 26074963

[bib15] Clemmons A. W. Lindsay S. A. Wasserman S. A., 2015 An effector peptide family required for *Drosophila* Toll-mediated immunity. PLoS Pathog. 11: e1004876. 25915418 10.1371/journal.ppat.1004876 PMC4411088

[bib16] Cohen L. Moran Y. Sharon A. Segal D. Gordon D., 2009 Drosomycin, an innate immunity peptide of *Drosophila melanogaster*, interacts with the fly voltage-gated sodium channel. J. Biol. Chem. 284: 23558–23563. 19574227 10.1074/jbc.M109.023358 PMC2749130

[bib17] Dalla Libera A. L. Regner A. de Paoli J. Centeraro L. Martins T. T., 2011 IL-6 polymorphisms associated with fatal outcome in patients with severe traumatic brain injury. Brain Inj. 25: 365–369. 21314275 10.3109/02699052.2011.556107

[bib18] Dardiotis E. Fountas K. N. Dardioti M. Xiromerisiou G. Kapsalaki E., 2010 Genetic association studies in patients with traumatic brain injury. Neurosurg. Focus 28: E9. 10.3171/2009.10.FOCUS09215 20043724

[bib19] Davis L. M. Pauly J. R. Readnower R. D. Rho J. M. Sullivan P. G., 2008 Fasting is neuroprotective following traumatic brain injury. J. Neurosci. Res. 86: 1812–1822. 18241053 10.1002/jnr.21628

[bib20] De Gregorio E. Spellman P. T. Rubin G. M. Lemaitre B., 2001 Genome-wide analysis of the *Drosophila* immune response by using oligonucleotide microarrays. Proc. Natl. Acad. Sci. USA 98: 12590–12595. 11606746 10.1073/pnas.221458698 PMC60098

[bib21] De Gregorio E. Spellman P. T. Tzou P. Rubin G. M. Lemaitre B., 2002 The Toll and Imd pathways are major regulators of the immune response in *Drosophila*. EMBO J. 21: 2568–2579. 12032070 10.1093/emboj/21.11.2568 PMC126042

[bib22] Deng-Bryant Y. Prins M. L. Hovda D. A. Harris N. G., 2011 Ketogenic diet prevents alternations in brain metabolism in young but not adult rats after traumatic brain injury. J. Neurotrauma 28: 1813–1825. 21635175 10.1089/neu.2011.1822 PMC3172875

[bib23] Dhandapani S. Manju D. Sharma B. Mahapatra A., 2012 Prognostic significance of age in traumatic brain injury. J. Neurosci. Rural Pract. 3: 131–135. 22865961 10.4103/0976-3147.98208 PMC3409980

[bib24] Djordjevic J. Sabbir M. G. Albensi B. C., 2016 Traumatic brain injury as a risk factor for Alzheimer’s disease: is inflammatory signaling a key player? Curr. Alzheimer Res. 13: 730–738. 26899581 10.2174/1567205013666160222110320

[bib25] Dobin A. Davis C. A. Schlesinger F. Drenkow J. Zaleski C., 2013 STAR: ultrafast universal RNA-seq aligner. Bioinformatics 29: 15–21. 23104886 10.1093/bioinformatics/bts635 PMC3530905

[bib26] Ekengren S. Hultmark D., 2001 A family of Turandot-related genes in the humoral stress response of *Drosophila*. Biochem. Biophys. Res. Commun. 284: 998–1003. 11409894 10.1006/bbrc.2001.5067

[bib27] Failla M. D. Kumar R. G. Peitzman A. B. Conley Y. P. Ferrell R. E., 2015 Variation in the BDNF gene interacts with age to predict mortality in a prospective, longitudinal cohort with severe TBI. Neurorehabil. Neural Repair 29: 234–246. 25063686 10.1177/1545968314542617 PMC4305354

[bib28] Failla M. D. Conley Y. P. Wagner A. K., 2016 Brain-derived neurotrophic factor (BDNF) in traumatic brain injury-related mortality: interrelationships between genetics and acute system and central nervous system BDNF profiles. Neurorehabil. Neural Repair 30: 83–93. 25979196 10.1177/1545968315586465 PMC4644728

[bib29] Feighery L. Smyth A. Keely S. Baird A. W. O’Connor W. T., 2008 Increased intestinal permeability in rats subjected to traumatic frontal lobe percussion brain injury. J. Trauma 64: 131–138. 18188111 10.1097/TA.0b013e3181568d9f

[bib30] Ferrandon D. Imler J. L. Hetru C. Hoffmann J. A., 2007 The *Drosophila* systemic immune response: sensing and signaling during bacterial and fungal infections. Nat. Rev. Immunol. 7: 862–874. 17948019 10.1038/nri2194

[bib31] Friess S. H. Ichord R. N. Ralston J. Ryall K. Halfaer M. A., 2009 Repeated traumatic brain injury affects composite cognitive function in piglets. J. Neurotrauma 26: 1111–1121. 19275468 10.1089/neu.2008.0845 PMC2848948

[bib32] Gadani S. P. Walsh J. T. Lukens J. R. Kipnis J., 2015 Dealing with danger in the CNS: the response of the immune system to injury. Neuron 87: 47–62. 26139369 10.1016/j.neuron.2015.05.019 PMC4491143

[bib33] Garringer J. A. Niyonkuru C. McCullough E. H. Loucks T. Dixon C. E., 2013 Impact of aromatase genetic variation on hormone levels and global outcome after severe TBI. J. Neurotrauma 30: 1415–1425. 23540392 10.1089/neu.2012.2565 PMC3741419

[bib34] Griesdale D. E. Tremblay M. H. McEwen J. Chittock D. R., 2009 Glucose control and mortality in patients with severe traumatic brain injury. Neurocrit. Care 11: 311–316. 19636972 10.1007/s12028-009-9249-1

[bib35] Heiman A. Pallottie A. Heary R. F. Elkabes S., 2014 Toll-like receptors in central nervous system injury and disease: a focus on the spinal cord. Brain Behav. Immun. 42: 232–245. 25063708 10.1016/j.bbi.2014.06.203

[bib36] Hellewell S. C. Morganti-Kossmann M. C., 2012 Guilty molecules, guilty mind? The conflicting roles of the innate immune response to traumatic brain injury. Mediators Inflamm. 2012: 356494. 22701273 10.1155/2012/356494 PMC3373171

[bib37] Hosseini P. Tremblay A. Matthews B. F. Alkharouf N. W., 2010 An efficient annotation and gene-expression derivation toll for Illumina Solexa datasets. BMC Res. Notes 3: 183. 20598141 10.1186/1756-0500-3-183 PMC2908109

[bib38] Hu Y. C. Sun Q. Li W. Zhang D. D. Ma B., 2014 Biphasic activation of nuclear factor kappa B and expression of p65 and c-Rel after traumatic brain injury in rats. Inflamm. Res. 63: 109–115. 24146067 10.1007/s00011-013-0677-1

[bib39] Huang L. Coat J. S. Mohd-Yusof A. Yin Y. Assaad S., 2013 Tissue vulnerability is increased following repetitive mild traumatic brain injury in the rat. Brain Res. 1499: 109–120. 23276495 10.1016/j.brainres.2012.12.038

[bib40] Huang X. J. Li W. P. Lin Y. Feng J. F. Jia F. F., 2014 Blockage of the upregulation of voltage-gated sodium channel nav1.3 improves outcomes after experimental traumatic brain injury. J. Neurotrauma 31: 346–357. 24313291 10.1089/neu.2013.2899 PMC3922240

[bib41] Hukkelhoven C. W. Steinberg E. W. Rampen A. J. Farace E. Habbema J. D., 2003 Patient age and outcome following severe traumatic brain injury: an analysis of 5600 patients. J. Neurosurg. 99: 666–673. 14567601 10.3171/jns.2003.99.4.0666

[bib42] Jiang H. Lei R. Ding S. W. Zhu S., 2014 Skewer: a fast and accurate adapter trimmer for next-generation sequencing paired-end reads. BMC Bioinformatics 15: 182. 24925680 10.1186/1471-2105-15-182 PMC4074385

[bib43] Kanayama G. Takeda M. Niigawa H. Ikura Y. Tamii H., 1996 The effects of repetitive mild brain injury on cytoskeletal protein and behavior. Methods Find. Exp. Clin. Pharmacol. 18: 105–115. 8740242

[bib44] Katzenberger R. J. Loewen C. A. Wassarman D. R. Petersen A. J. Ganetzky B., 2013 A *Drosophila* model of closed head traumatic brain injury. Proc. Natl. Acad. Sci. USA 110: E4152–E4159. 24127584 10.1073/pnas.1316895110 PMC3816429

[bib45] Katzenberger R. J. Chtarbanova S. Rimkus S. A. Fischer J. A. Kaur G., 2015 a Death following traumatic brain injury in *Drosophila* is associated with intestinal barrier dysfunction. eLife 4: 04790. 10.7554/eLife.04790 PMC4377547 25742603

[bib46] Katzenberger R. J. Loewen C. A. Bockstruck R. T. Woods M. A. Ganetzky B., 2015 b A method to inflict closed head traumatic brain injury in *Drosophila*. J. Vis. Exp. 100: e52905. 10.3791/52905 PMC4544997 26168076

[bib47] Katzenberger R. J. Ganetzky B. Wassarman D. A., 2015 c The gut reaction to traumatic brain injury. Fly (Austin) 9: 68–74. 26291482 10.1080/19336934.2015.1085623 PMC5019014

[bib48] Krishnamurthy K. Laskowitz D. T., 2016 Cellular and molecular mechanisms of secondary neuronal injury following traumatic brain injury, chapter 5, in Translational Research in Traumatic Brain Injury, edited by Laskowitz D. Grant G. CRC Press/Taylor and Francis Group, Boca Raton. 26583177

[bib49] Kurata S., 2014 Peptidoglycan recognition proteins in *Drosophila* immunity. Dev. Comp. Immunol. 42: 36–41. 23796791 10.1016/j.dci.2013.06.006 PMC3808481

[bib50] Lamiable O. Kellenberger C. Kemp C. Troxler L. Pelte N., 2016 Cytokine Diedel and a viral homologue suppress the IMD pathway in *Drosophila*. Proc. Natl. Acad. Sci. USA 113: 698–703. 26739560 10.1073/pnas.1516122113 PMC4725508

[bib51] Lemaitre B. Hoffmann J., 2007 The host defense of *Drosophila melanogaster*. Annu. Rev. Immunol. 25: 697–743. 17201680 10.1146/annurev.immunol.25.022106.141615

[bib52] Lemaitre B. Reichhart J. M. Hoffmann J. A., 1997 *Drosophila* host defense: differential induction of antimicrobial peptide genes after infection by various classes of microorganisms. Proc. Natl. Acad. Sci. USA 94: 14614–14619. 9405661 10.1073/pnas.94.26.14614 PMC25070

[bib53] Li B. Dewey C. N., 2011 RSEM: accurate transcript quantification from RNA-Seq data with or without a reference genome. BMC Bioinformatics 12: 323. 21816040 10.1186/1471-2105-12-323 PMC3163565

[bib54] Lu J. Goh S. J. Tng P. Y. Deng Y. Y. Ling E. A., 2009 Systemic inflammatory response following acute traumatic brain injury. Front. Biosci. 14: 3795–3813. 10.2741/3489 19273311

[bib55] Mackay T. F. Richards S. Stone E. A. Barbadilla A. Ayroles J. F., 2012 The *Drosophila melanogaster* genetic reference panel. Nature 482: 173–178. 22318601 10.1038/nature10811 PMC3683990

[bib56] Masel B. E. Dewitt D. S., 2010 Traumatic brain injury: a disease process, not an event. J. Neurotrauma 27: 1529–1540. 20504161 10.1089/neu.2010.1358

[bib57] Meehan W. P. III Zhang J. Minnix R. Whalen M. J., 2012 Increasing recovery time between injuries improves cognitive outcome after repetitive mild concussive brain injuries in mice. Neurosurgery 71: 885–891. 22743360 10.1227/NEU.0b013e318265a439 PMC5815628

[bib58] Mychasiuk R. Hehar H. van Waes L. Esser M. J., 2015 Diet, age, and prior injury status differentially alter behavior outcomes following concussion in rats. Neurobiol. Dis. 73: 1–11. 25270295 10.1016/j.nbd.2014.09.003

[bib59] Natale J. E. Ahmed F. Cernak I. Stoica B. Faden A. I., 2003 Gene expression profile changes are commonly modulated across models and species after traumatic brain injury. J. Neurotrauma 20: 907–927. 14588109 10.1089/089771503770195777

[bib60] Owusu-Ansah E. Perrimon N., 2015 Stress signaling between organs in metazoa. Annu. Rev. Cell Dev. Biol. 31: 497–522. 26393775 10.1146/annurev-cellbio-100814-125523

[bib61] Papai G. Weil P. A. Schultz P., 2011 New insights into the function of transcription factor TFIID from recent structural studies. Curr. Opin. Genet. Dev. 21: 219–224. 21420851 10.1016/j.gde.2011.01.009 PMC3081712

[bib62] Petersen A. J. Rimkus S. A. Wassarman D. A., 2012 ATM kinase inhibition in glial cells activates the innate immune response and causes neurodegeneration in *Drosophila*. Proc. Natl. Acad. Sci. USA 109: E656–E664. 22355133 10.1073/pnas.1110470109 PMC3306708

[bib63] Prins M. L. Matsumoto J. H., 2014 The collective therapeutic potential of cerebral ketone metabolism in traumatic brain injury. J. Lipid Res. 55: 2450–2457. 24721741 10.1194/jlr.R046706 PMC4242438

[bib64] Prins M. L. Fujima L. S. Hovda D. A., 2005 Age-dependent reduction of cortical contusion volume by ketones after traumatic brain injury. J. Neurosci. Res. 82: 413–420. 16180224 10.1002/jnr.20633

[bib65] Rajan A. Perrimon N., 2012 *Drosophila* cytokine unpaired 2 regulated physiological homeostasis by remotely controlling insulin secretion. Cell 151: 123–137. 23021220 10.1016/j.cell.2012.08.019 PMC3475207

[bib66] Redell J. B. Moore A. N. Grill R. J. Johnson D. Zhao J., 2013 Analysis of functional pathways altered after mild traumatic brain injury. J. Neurotrauma 30: 752–764. 22913729 10.1089/neu.2012.2437 PMC3653386

[bib67] Rivest S., 2009 Regulation of innate immune responses in the brain. Nat. Rev. Immunol. 9: 429–439. 19461673 10.1038/nri2565

[bib68] Robinson M. D. McCarthy D. J. Smyth G. K., 2010 edgeR: a bioconductor package for differential expression analysis of digital gene expression data. Bioinformatics 26: 139–140. 19910308 10.1093/bioinformatics/btp616 PMC2796818

[bib69] Smith D. H. Johnson V. E. Stewart W., 2013 Chronic neuropathologies of single and repetitive TBI: substrates of dementia? Nat. Rev. Neurol. 9: 211–221. 23458973 10.1038/nrneurol.2013.29 PMC4513655

[bib70] Sperry J. L. Zolin S. Zuckerbraun B. S. Vodovotz Y. Namas R., 2014 X chromosome-linked IRAK-1 polymorphism is a strong predictor of multiple organ failure and mortality postinjury. Ann. Surg. 260: 698–703. 25203887 10.1097/SLA.0000000000000918 PMC4159729

[bib71] Stec W. Vidal O. Zeidler M. P., 2013 *Drosophila* SOCS36E negatively regulates JAK/STAT pathway signaling via two separable mechanisms. Mol. Biol. Cell 24: 3000–3009. 23885117 10.1091/mbc.E13-05-0275 PMC3771960

[bib72] Stocchetti N. Zanier E. R., 2016 Chronic impact of traumatic brain injury on outcome and quality of life: a narrative review. Crit. Care 20: 148. 27323708 10.1186/s13054-016-1318-1 PMC4915181

[bib73] Susman M. DiRusso S. M. Sullivan T. Risucci D. Nealon P., 2002 Traumatic brain injury in the elderly: increased mortality and worse functional outcome at discharge despite lower injury severity. J. Trauma 53: 219–223. 12169925 10.1097/00005373-200208000-00004

[bib74] Wang H.-C. Sun S. C.-F. Chen H. Chen M.-S. Shen G., 2014 Where are we in the modeling of traumatic brain injury? Models complicated by secondary brain insults. Brain Inj. 28: 1491–1503. 25111457 10.3109/02699052.2014.943288

[bib75] Wang X. Dong Y. Han X. Qi X. Q. Huang C. G., 2013 Nutritional support for patients sustaining traumatic brain injury: a systematic review and meta-analysis of prospective studies. PLoS One 8: e58838. 23527035 10.1371/journal.pone.0058838 PMC3602547

[bib76] Weil Z. M. Gaier K. R. Karelina K., 2014 Injury timing alters metabolic, inflammatory and functional outcomes following repeated mild traumatic brain injury. Neurobiol. Dis. 70: 108–116. 24983210 10.1016/j.nbd.2014.06.016

[bib77] White T. E. Ford G. D. Surles-Zeiger M. C. Gates A. S. Laplaca M. C., 2013 Gene expression patterns following unilateral traumatic brain injury reveals a local pro-inflammatory and remote anti-inflammatory response. BMC Genomics 14: 282. 23617241 10.1186/1471-2164-14-282 PMC3669032

[bib78] Wong Y. H. Wu C. C. Lai J. C. Chen K. Y. Jheng B. R., 2016 Temporal genetic modifications after controlled cortical impact—understanding traumatic brain injury through a systematic network approach. Int. J. Mol. Sci. 17: 216. 26861311 10.3390/ijms17020216 PMC4783948

[bib79] Woodcock T. Morganti-Kossmann M. C., 2013 The role of markers of inflammation in traumatic brain injury. Front. Neurol. 4: 18. 23459929 10.3389/fneur.2013.00018 PMC3586682

[bib80] Zaidman-Rémy A. Hervé M. Poidevin M. Pili-Floury S. Kim M. S., 2006 The *Drosophila* amidase PGRP-LB modulates the immune response to bacterial infection. Immunity 24: 463–473. 16618604 10.1016/j.immuni.2006.02.012

