# Supplementary material for: Age and Diet Affect Genetically Separable Secondary Injuries that Cause Acute Mortality Following Traumatic Brain Injury in Drosophila
Source: G3 (Bethesda). 2016 Oct 17;6(12):4151–66. doi: 10.1534/g3.116.036194 (PMC5144983; doi:10.1534/g3.116.036194)
Supplement: Supplemental Material [file supp_6_12_4151__index.html]

Age and Diet Affect Genetically Separable Secondary Injuries that Cause Acute Mortality Following Traumatic Brain Injury in Drosophila — Supplemental Material 

# Age and Diet Affect Genetically Separable Secondary Injuries that Cause Acute Mortality Following Traumatic Brain Injury in *Drosophila*

## Supplemental Material for Wassarman *et al.*, 2016

**Files in this Data Supplement:**

- Table S1 - *P*-values, one-tailed *t* test comparison of the MI24 between the indicated conditions for different fly lines. (.pdf, 46 KB)
- Table S2 - Genes up-regulated following primary injuries. (.pdf, 113 KB)
- Table S3 - Genes down-regulated following primary injuries. (.pdf, 57 KB)
- Table S4 - Analysis of absolute expression after primary injuries. (.pdf, 46 KB)
- Table S5 - Primers for RT-qPCR. (.pdf, 46 KB)
