# Supplementary material for: Age and Diet Affect Genetically Separable Secondary Injuries that Cause Acute Mortality Following Traumatic Brain Injury in Drosophila
Source: G3 (Bethesda). 2016 Oct 17;6(12):4151–66. doi: 10.1534/g3.116.036194 (PMC5144983; doi:10.1534/g3.116.036194)
Supplement: Supplemental Material [file supp_g3.116.036194_TableS1.pdf]

Table S1. *P*-values, one-tailed *t* test comparison of the MI_{24} between the indicated conditions for different fly lines*

Fly line	Age: younger vs. older				Diet: water vs. food				Inter-injury interval: 5 min vs. 2 h			
	Water	Food	Water	Food	Younger	Older	Younger	Older	Younger	Older	Younger	Older
	5 min	5 min	2 h	2 h	5 min	5 min	2 h	2 h	Water	Water	Food	Food
w^{1118}	<0.0001	<0.0001	<0.0001	<0.0001	<0.0001	0.0010	<0.0001	<0.0001	0.3508	0.4990	0.0034	0.0058
RAL26	0.1963	0.0061	0.3174	0.0035	0.0139	0.0021	0.0304	0.0020	0.7369	0.5790	0.1900	0.3679
RAL38	0.2031	0.3538	0.3416	0.1803	0.0549	0.3929	0.0503	0.1373	0.7532	0.7811	0.5653	0.7133
RAL45	0.6320	0.0049	0.0551	0.0781	0.2659	0.1177	0.1300	0.1247	0.3392	0.2792	0.8977	0.3934
RAL69	0.0002	0.0012	<0.0001	0.0004	0.0043	0.1018	0.0010	<0.0001	0.5593	0.1638	0.0184	0.0128
RAL73	0.0834	0.0028	0.1343	0.0256	0.1455	0.0533	0.0270	0.0904	0.4795	0.7333	0.6931	0.8054
RAL83	0.0330	0.0090	0.0270	0.0225	0.0113	0.0028	0.0371	0.0152	0.7986	0.7780	0.3506	0.3268
RAL85	0.0155	0.0300	0.3074	0.0034	0.0869	0.0043	0.0937	0.0078	0.1575	0.3876	0.8500	0.2181
RAL88	0.0153	0.0081	0.0348	0.0848	0.0380	0.0103	0.5992	0.1154	0.1674	0.9920	0.6244	0.8821
RAL91	0.1075	0.0451	0.2438	0.3528	0.0466	0.4041	0.0095	0.1183	0.8650	0.2124	0.7307	0.3825
RAL93	0.0143	0.0002	0.0012	0.0144	0.0161	0.0478	0.0311	0.0969	0.0945	0.8076	0.6663	0.8737
RAL105	0.0363	0.0020	0.0916	0.0135	0.0164	0.0270	0.0075	0.0193	0.6779	0.5061	0.2041	0.3739
RAL161	0.7708	0.0176	0.0030	0.3417	0.1207	0.0012	0.0003	0.0663	0.2777	0.2482	0.4552	0.4674
RAL381	0.8773	0.3571	0.6432	0.2828	0.1118	0.0260	0.2879	0.0190	0.8555	0.8478	0.5097	0.3830
RAL382	0.0289	0.0018	0.3809	0.0469	0.0026	0.0030	0.6542	0.0857	0.0687	0.9064	0.1397	0.5484
RAL383	0.0126	0.1500	0.0838	0.0517	0.2087	0.7634	0.0456	0.0730	0.9907	0.1064	0.8804	0.6491
RAL391	0.0173	0.0046	0.0004	0.3162	0.0036	0.0017	0.0182	0.0017	0.1824	0.1244	0.3545	0.1295
RAL392	0.1908	0.0751	0.1720	0.0101	0.0003	0.0174	0.0004	0.0086	0.8641	0.4116	0.0544	0.8856
RAL409	0.0497	<0.0001	0.0311	<0.0001	<0.0001	0.0123	0.0029	0.0044	0.6949	0.8808	0.7296	0.0343
RAL427	0.6754	0.2373	0.5089	0.3908	0.0037	0.0478	0.0279	0.0481	0.7945	0.3779	0.2721	0.2669
RAL439	0.0038	0.0203	0.0437	0.0006	0.4820	0.9304	0.6433	0.0132	>0.9999	0.0502	>0.9999	0.8521
RAL440	0.0083	0.4377	0.2907	0.0411	0.1103	0.8106	0.4448	0.0442	0.1808	0.0533	0.5588	0.2469
RAL441	0.0504	0.0547	0.0520	0.0447	0.0435	0.0175	0.0732	0.4676	0.6225	0.2028	0.3631	0.7676
RAL443	0.0042	0.1248	0.1576	0.0285	0.0201	0.4012	0.1330	0.0560	0.4458	0.9111	0.6153	0.0997
RAL853	0.1979	0.2110	0.2567	0.1022	0.3482	0.0067	0.4417	0.1338	0.9104	0.6918	0.9303	0.0445
RAL859	0.3341	0.2312	0.2792	0.2111	0.3642	0.0391	0.2340	0.3329	0.6627	0.8073	0.7467	0.1758
RAL882	0.0612	<0.0001	0.0037	0.0231	0.0019	0.0045	0.0021	0.0383	0.7988	0.8090	0.0397	0.0178
RAL892	0.0006	0.0097	0.0046	0.0003	0.0292	0.0074	0.0009	0.0105	0.8112	0.6062	0.4100	0.0513
RAL897	0.0073	0.0674	0.0006	0.0007	0.0138	0.0005	0.0146	0.0009	0.9864	0.0776	0.0635	0.0226
RAL900	0.0420	0.0012	0.0020	0.0728	0.0291	0.0362	0.0363	0.1661	0.6686	0.4945	>0.9999	0.1099
RAL911	0.0233	0.0025	0.5588	0.0093	0.0308	<0.0001	0.1266	0.0003	0.5811	0.2623	0.3256	0.2121

*Analysis of the data presented in Figures 3A and B. *P*-values in this table were used to generate Table 1.
Green shading: significantly increased MI_{24} ($P<0.05$)
Red shading: significantly decreased MI_{24} ($P<0.05$)
