# Supplementary material for: Age and Diet Affect Genetically Separable Secondary Injuries that Cause Acute Mortality Following Traumatic Brain Injury in Drosophila
Source: G3 (Bethesda). 2016 Oct 17;6(12):4151–66. doi: 10.1534/g3.116.036194 (PMC5144983; doi:10.1534/g3.116.036194)
Supplement: Supplemental Material [file supp_g3.116.036194_TableS2.pdf]

Table S2. Genes up-regulated following primary injuries (see Figure 4A)

a (17)	b (33)	c (91)	d (175)	e (6)	f (9)	g (9)
CG10232	CG12826	CR32864	Act42A	CG11459	CG14695	CG13311
CG11741	CG13305	CR42652	Ag5r2	Diedel	CG31775	CG15067
CG34162	CG13461	Arc1	CG10086	TotA	CG42586	CG16704
CG4725	CG14050	CG10420	CG10163	TotC	CG4757	CG16712
Cp15	CG14743	CG11069	CG10300	TotM	CG5791	CG32023
Cpr47Eb	CG15332	CG11425	CG10431	TotX	Ccp84Ad	CG9400
Cyp6a19	CG18477	CG12520	CG10650		Tsf1	mthl2
Fad2	CG2901	CG12655	CG10834		lmd	rgn
Obp19c	CG31780	CG12880	CG10898		nimB3	spz5
Yp2	CG3280	CG13117	CG10962			
Yp3	CG34265	CG13618	CG11034			
1-Dec	CG34426	CG13795	CG11318			
dhd	CG43146	CG13841	CG11529			
lectin-24A	CG8908	CG13894	CG11898			
vanin-like	CG9701	CG14661	CG12009			
wisp	CR43465	CG14906	CG1246			
yl	CR43475	CG15043	CG12795			
	CR43809	CG1673	CG13004			
	CR44055	CG16836	CG13386			
	Cyp4p3	CG2004	CG13559			
	Ilp8	CG30002	CG14500			
	Kif19A	CG30154	CG14869			
	MESK4	CG3097	CG14879			
	Obp56e	CG31121	CG15044			
	Pvf2	CG3117	CG15126			
	Tep1	CG31769	CG15423			
	TotB	CG31809	CG15745			
	cutlet	CG31869	CG17104			
	ninaD	CG33460	CG1806			
	pre-mod(mdg4)-AD	CG33993	CG18635			
	pre-mod(mdg4)-X	CG3505	CG18744			
	stet	CG3604	CG18745			
	tj	CG42259	CG18748			
		CG42662	CG18769			
		CG42807	CG2065			
		CG42808	CG2865			
		CG42821	CG30424			
		CG42868	CG31004			
		CG43091	CG31259			
		CG43093	CG31324			
		CG4374	CG3165			
		CG44140	CG32053			
		CG44141	CG32137			
		CG5493	CG3259			
		CG5778	CG33223			
		CG6188	CG3332			
		CG6357	CG33511			
		CG6361	CG34165			
		CG6426	CG34194			
		CG6675	CG34198			
		CG7017	CG34301			
		CG7637	CG3690			
		CG8046	CG3884			
		CG8738	CG3921			

		CG9411	CG42565			
		CG9759	CG42666			
		CG9928	CG42709			
		CR40677	CG42825			
		CR41602	CG43773			
		CR43474	CG43774			
		Ccp84Aa	CG4781			
		Cpr47Ef	CG5011			
		Def	CG5290			
		Edg91	CG5346			
		GstD5	CG5399			
		GstE1	CG5525			
		Idgf1	CG5767			
		ImpL1	CG5770			
		Irc	CG5810			
		LKR	CG5835			
		MtnD	CG5869			
		Nmdmc	CG6296			
		Nop56	CG6701			
		Nop60B	CG6933			
		Obp99d	CG6967			
		Pk1r	CG7142			
		SPE	CG7255			
		Ser7	CG7702			
		Spargel	CG8026			
		Tig	CG8083			
		Tsp42El	CG8093			
		Ugh2	CG8258			
		capt	CG8785			
		nop5	CG9171			
		pll	CG9555			
		rpr	CG9568			
		scaf	CG9737			
		skpC	CR43263			
		spirit	CR43468			
		tnc	CR43920			
		yellow-e	CR44192			
			Cortactin			
			Cpr76Bd			
			Cralbp			
			Dhap-at			
			Drsl2			
			Drsl3			
			Ect3			
			Fancl			
			Fim			
			G-ialpha65A			
			GEFmeso			
			Gclm			
			GlcT-1			
			Gli			
			Gr98a			
			GstE10			
			HP1D3csd			
			Hip1			
			Hop			

			Hsc70Cb			
			Hsp27			
			Hsp70Bc			
			Hsp83			
			ImpL3			
			Inx7			
			Ipk2			
			Jon25Biii			
			Jon65Ai			
			Jon66Ci			
			Jon99Cii			
			Jon99Ciii			
			Jon99Fi			
			Jon99Fii			
			LamC			
			MRE23			
			Meltrin			
			Oatp33Eb			
			PGRP-LF			
			PGRP-SC2			
			Pa1			
			Pak3			
			Pu			
			Pvr			
			Rac2			
			Rcd2			
			RhoGAP15 B			
			RhoL			
			Sfp79B			
			Sip1			
			Spn42Da			
			Tehao			
			Tsp2A			
			Tsp42Eb			
			Tsp42Ec			
			Tsp42Ed			
			Tsp42Eg			
			Whamy			
			alt			
			betaInt-nu			
			betaTub60D			
			dl			
			dome			
			dos			
			dream			
			eloF			
			ena			
			fit			
			insc			
			lace			
			luna			
			mthl14			
			mthl9			
			mys			
			pain			

			pcl				
			pot				
			prominin-like				
			puc				
			rho-4				
			sda				
			tamo				
			th				
			topi				
			wdp				

Table S2 continued. Genes up-regulated following primary injuries (see Figure 4A)

h (1)	I (21)	j (82)	k (1)	l (2)	m (1)	n (67)	o (57)
Yp1	CG10140	ALiX	Ccp84Ab	PGRP-SB1	CG7298	AnnIX	AttA
	CG10625	Act5C		alphaPS5		AttD	AttB
	CG14204	Arc2				CG10126	AttC
	CG14961	CG10103				CG10337	CG10182
	CG15282	CG10211				CG10663	CG10332
	CG18563	CG10527				CG12814	CG13324
	CG30098	CG10641				CG1299	CG13422
	CG32302	CG1129				CG13075	CG13641
	CG4194	CG12112				CG13215	CG13905
	CG42262	CG12560				CG13323	CG14957
	CG5630	CG13024				CG13325	CG15065
	CR31514	CG13177				CG14205	CG15829
	CR43654	CG13321				CG14219	CG16713
	Hsp23	CG13482				CG14499	CG16772
	Hsp26	CG13510				CG14567	CG16978
	Hsp70Ba	CG13511				CG15021	CG18067
	Hsp70Bb	CG14907				CG15097	CG18557
	Hsp70Bbb	CG15046				CG15155	CG30026
	PGRP-LB	CG15784				CG15673	CG33470
	alphaPS4	CG16718				CG1572	CG34054
	upd2	CG16775				CG2217	CG43085
		CG17278				CG30151	CG43165
		CG17760				CG34043	CG43175
		CG18530				CG34349	CG43202
		CG1890				CG42561	CG43236
		CG32107				CG4269	CG5550
		CG32284				CG42740	CG6639
		CG33258				CG42867	CG9733
		CG33337				CG43095	CG9989
		CG33468				CG43194	CecA2
		CG3348				CG43210	CecB
		CG3814				CG43348	CecC
		CG4199				CG4367	Dpt
		CG4267				CG5191	DptB
		CG43064				CG5246	Dro
		CG5773				CG6429	Drs
		CG5909				CG8791	Ets21C
		CG6553				CG9631	Fst
		CG7720				CecA1	Gadd45
		CG8192				Cht9	GstD2
		CG8620				Ddc	IM1
		CG8952				Fas3	IM10

		CG8965				Hsp68	IM14
		CG9447				Hsp70Aa	IM18
		CG9452				Hsp70Ab	IM2
		CHMP2B				Mmp1	IM23
		CR43334				Myo31DF	IM3
		CR44179				Naam	IM4
		CalpB				Rel	Listericin
		Cct5				Sp212	Mtk
		Cctgamma				Sp7	PGRP-SA
		Chmp1				Spn28Dc	Socs36E
		Cht4				Swim	Spn88Eb
		CrebA				Tep4	Tep2
		Ets98B				Timp	Uro
		Hus1-like				Tsf3	edin
		Idgf3				Tsp42Ef	nimB1
		Jra				Wsck	
		Lsd-1				b6	
		Mvl				cher	
		NijA				jhamt	
		Orct2				pirk	
		PGRP-SD				ple	
		Phk-3				pncr016:2R	
		QC				spz3	
		SH3PX1				upd3	
		Spn27A				yellow-b	
		Spn55B					
		T-cp1					
		Tcp-1eta					
		Tre1					
		aay					
		cact					
		mthl5					
		nec					
		p38c					
		rho					
		scb					
		schlank					
		spz					
		viaf					
		vir-1					
