# Supplementary material for: Age and Diet Affect Genetically Separable Secondary Injuries that Cause Acute Mortality Following Traumatic Brain Injury in Drosophila
Source: G3 (Bethesda). 2016 Oct 17;6(12):4151–66. doi: 10.1534/g3.116.036194 (PMC5144983; doi:10.1534/g3.116.036194)
Supplement: Supplemental Material [file supp_g3.116.036194_TableS3.pdf]

Table S3. Genes down-regulated following primary injuries (see Figure 4B)

a (28)	b (83)	c (20)	d (77)	e (3)	f (4)	g (8)	h (0)
CG11380	Ance-4	CG13188	18w	CG18765	CG4830	CG10051	
CG12256	CG10936	CG14120	CG10131	CG3106	CG6901	CG1946	
CG13813	CG11192	CG15253	CG10361	Npc2c	CG9465	CG33966	
CG14406	CG11236	CG17374	CG10560		ninaD	CG34291	
CG14502	CG11407	CG18179	CG10570			CG5107	
CG17298	CG11594	CG32984	CG13404			CG7025	
CG2444	CG11893	CG33012	CG13670			CG8147	
CG2650	CG12057	CG34316	CG13856			Jon65Aii	
CG31089	CG12766	CG3775	CG14400				
CG34116	CG12970	CG4914	CG1461				
CG34461	CG13078	CG5999	CG1561				
CG4017	CG13160	CG6300	CG16727				
CG42759	CG13285	CG7402	CG16798				
CG6012	CG13722	CR43452	CG17803				
CG8093	CG14259	Cyp304a1	CG17999				
CG8534	CG14356	Jon66Ci	CG1887				
CG9826	CG14523	Sox100B	CG2736				
CG9826	CG14990	Ts	CG31053				
CR43866	CG15221	mira	CG31106				
Cht5	CG15353	nau	CG31202				
Jon99Ci	CG15534		CG31205				
LysB	CG17147		CG31207				
LysC	CG17192		CG31743				
Rab9D	CG18302		CG3239				
CR32865	CG18585		CG32647				
inaF-D	CG31076		CG34120				
l(3)mbn	CG31703		CG34313				
nompA	CG31823		CG3902				
	CG32037		CG42502				
	CG32335		CG4335				
	CG3264		CG4500				
	CG3326		CG4822				
	CG33306		CG4962				
	CG33307		CG5508				
	CG34166		CG5973				
	CG4000		CG6034				
	CG4053		CG6067				
	CG42728		CG6074				
	CG42741		CG6188				
	CG43104		CG6870				
	CG43196		CG6908				
	CG43997		CG6910				
	CG44173		CG7079				
	CG4476		CG8112				
	CG4734		CG8654				
	CG4842		CG8854				
	CG5070		CG9317				
	CG6793		CR43730				
	CG6834		CR44024				
	CG7542		CR44133				
	CG7916		Cyp18a1				
	CG7953		Cyp28d1				
	CG8562		Cyp6a8				
	CG8708		DAT				
	CG8997		Eip71CD				
	CG9095		Gk				
	CG9150		Lin29				
	CG9825		List				
	CG9993		Ork1				
	CR43632		Reck				
	CR44106		Sema-2a				
	Cht7		Smvt				
	Cpr72Ec		St1				
	Diedel3		alpha-Est8				

	Elo68alpha		cd				
	Hex-C		disco-r				
	HisCl1		ds				
	Hml		dysc				
	Jon44E		fusl				
	Jon99Fii		gl				
	Npc2d		grh				
	NtR		opa				
	Obp83cd		pnr				
	PGRP-SC1b		prom				
	RNaseP:RNA		pug				
	RhoGEF4		shd				
	alpha-Est7		trpl				
	fj						
	iotaTry						
	mag						
	ms(3)K81						
	nimB4						
	thetaTry						

Table S3 continued. Genes down-regulated following primary injuries (see Figure 4B)

**i (29)**	**j (2)**	**k (6)**	**l (2)**	**m (0)**	**n (11)**	**o (4)**
CG11796	CG9119	CG15533	CG34217		CG13283	CG15263
CG13082	stl	CG31091	pre-mod(mdg4)-V		CG16756	CG4950
CG14105		CG9463			CG34211	Ser8
CG14823		CG9466			CG4408	nimC2
CG15414		CG9468			CG7224	
CG2070		Jon25Bii			Cyp4d1	
CG42369					Cyp6g1	
CG8665					Cyp6t1	
CG9259					Cyt-b5-r	
Cyp12d1-p					Ugt37b1	
Cyp313a1					alpha-Est2	
Cyp6a2						
Cyp6w1						
GstZ2						
Ir75a						
Ir75b						
Obp83ef						
Rfabg						
Sam-S						
Sodh-1						
Sr-CI						
alpha-Est1						
eater						
hgo						
lectin-24Db						
lectin-33A						
nimC1						
pon						
sxe2						
