# Supplementary material for: Age and Diet Affect Genetically Separable Secondary Injuries that Cause Acute Mortality Following Traumatic Brain Injury in Drosophila
Source: G3 (Bethesda). 2016 Oct 17;6(12):4151–66. doi: 10.1534/g3.116.036194 (PMC5144983; doi:10.1534/g3.116.036194)
Supplement: Supplemental Material [file supp_g3.116.036194_TableS4.pdf]

Table S4. Analysis of absolute expression after primary injuries (see Figures 6-10)

Gene	Condition	U	Ave. 5, 30 min	Ave. 1-8 h	24 h
AttC	Younger water	0.01811	0.01335	0.31712	0.09389
	Younger food	0.00356	0.00571	0.14150	0.03640
	Older water	0.02073	0.02804	0.48416	0.32109
	Older food	0.01940	0.02332	0.51004	0.11844
Def	Younger water	0.02700	0.02283	0.04098	0.01017
	Younger food	0.01684	0.01379	0.01990	0.01653
	Older water	0.03041	0.03778	0.04731	0.01950
	Older food	0.03331	0.02555	0.04152	0.02481
DiptB	Younger water	0.04771	0.03177	0.72139	0.28991
	Younger food	0.02206	0.01678	0.34108	0.10051
	Older water	0.26805	0.09390	1.07247	0.86026
	Older food	0.16054	0.08655	1.41704	0.50286
Dro	Younger water	0.00395	0.00280	0.02627	0.02186
	Younger food	0.00182	0.00186	0.00970	0.00807
	Older water	0.01895	0.00529	0.04528	0.05708
	Older food	0.00482	0.00506	0.04150	0.01532
Drs	Younger water	0.16450	0.07975	0.56798	0.30750
	Younger food	0.07441	0.05161	0.28453	0.09232
	Older water	0.19600	0.10942	0.48031	0.44133
	Older food	0.08088	0.09399	0.28505	0.13775
Mtk	Younger water	0.18203	0.07183	1.49507	0.81798
	Younger food	0.04538	0.04203	0.79698	0.21855
	Older water	0.22155	0.23419	1.75238	1.56424
	Older food	0.25010	0.21467	1.78984	0.88409
Spz	Younger water	0.00605	0.01304	0.01710	0.01114
	Younger food	0.00683	0.00885	0.01710	0.01015
	Older water	0.01017	0.00992	0.01967	0.01667
	Older food	0.00695	0.00974	0.01433	0.00532
Spz3	Younger water	0.00121	0.00137	0.00197	0.00115
	Younger food	0.00117	0.00074	0.00172	0.00044
	Older water	0.00107	0.00110	0.00137	0.00139
	Older food	0.00044	0.00028	0.00046	0.00015
Spz4	Younger water	0.00012	0.00012	0.00009	0.00015
	Younger food	0.00006	0.00006	0.00005	0.00004
	Older water	0.00011	0.00007	0.00006	0.00007
	Older food	0.00008	0.00003	0.00004	0.00001
Spz5	Younger water	0.00045	0.00048	0.00062	0.00113
	Younger food	0.00138	0.00087	0.00132	0.00087
	Older water	0.00095	0.00071	0.00097	0.00090
	Older food	0.00042	0.00038	0.00048	0.00041
Spz6	Younger water	0.00016	0.00009	0.00008	0.00010
	Younger food	0.00012	0.00007	0.00009	0.00005
	Older water	0.00007	0.00005	0.00005	0.00008
	Older food	0.00007	0.00005	0.00004	0.00001
TotA	Younger water	0.26589	0.18881	2.10391	4.90903
	Younger food	0.05971	0.14241	2.51822	3.59852
	Older water	0.70840	0.58190	1.56348	3.14638
	Older food	0.57195	0.83883	2.46208	5.61128
TotC	Younger water	0.19165	0.16589	1.64572	3.30117
	Younger food	0.10260	0.10011	1.24281	1.80559
	Older water	0.51662	0.37196	1.02307	1.81215
	Older food	0.47528	0.78237	1.61086	3.70676
TotM	Younger water	0.03971	0.04139	0.41040	2.43740
	Younger food	0.01268	0.01355	0.22135	0.22863

	Older water	0.03971	0.03920	0.37881	1.08753
	Older food	0.05992	0.05567	0.38428	0.56488
TotX	Younger water	0.01406	0.01166	0.09598	0.26438
	Younger food	0.00966	0.00842	0.09081	0.09873
	Older water	0.01406	0.01235	0.08305	0.18248
	Older food	0.03938	0.02426	0.07184	0.09369
Diedel	Younger water	0.00115	0.00090	0.02348	0.07839
	Younger food	0.00039	0.00027	0.01586	0.00794
	Older water	0.00104	0.00059	0.02299	0.05569
	Older food	0.00134	0.00038	0.01008	0.06347
Upd1	Younger water	0.00016	0.00014	0.00016	0.00019
	Younger food	0.00006	0.00005	0.00006	0.00005
	Older water	0.00019	0.00013	0.00018	0.00012
	Older food	0.00005	0.00003	0.00004	0.00003
Upd2	Younger water	0.00010	0.00012	0.00016	0.00008
	Younger food	0.00003	0.00007	0.00008	0.00003
	Older water	0.00012	0.00012	0.00018	0.00019
	Older food	0.00005	0.00011	0.00008	0.00003
Upd3	Younger water	0.00007	0.00008	0.00006	0.00007
	Younger food	0.00004	0.00004	0.00005	0.00002
	Older water	0.00012	0.00008	0.00014	0.00017
	Older food	0.00006	0.00007	0.00008	0.00002
TAF1	Younger water	0.00195	0.00117	0.00146	0.00191
	Younger food	0.00150	0.00071	0.00154	0.00079
	Older water	0.00172	0.00145	0.00253	0.00062
	Older food	0.00157	0.00090	0.00162	0.00048

Green shading: >2-fold change in expression for the average at 1-8 h post-injury (Ave. 1-8h) versus the average at 5 and 30 min post-injury (Ave. 5, 30 min) and at 24 h in uninjured flies (U). The data are derived from Figures 6-10.
