# Supplementary material for: Age and Diet Affect Genetically Separable Secondary Injuries that Cause Acute Mortality Following Traumatic Brain Injury in Drosophila
Source: G3 (Bethesda). 2016 Oct 17;6(12):4151–66. doi: 10.1534/g3.116.036194 (PMC5144983; doi:10.1534/g3.116.036194)
Supplement: Supplemental Material [file supp_g3.116.036194_TableS5.pdf]

Table S5. Primers for RT-qPCR

Gene	5’ Primer	3’ Primer
Attacin C (AttC)	CTGCACTGGACTACTCCCACATCA	CGATCCTGCGACTGCCAAAGATTG
Defensin (Def)	CCAGAGGATCATGTCCTGGTGCAT	ACTTGGAGAGTAGGTCGCATGTGG
Diedel	AGGGATCGTGCAACATCTTC	GCTGGTTCCTCCTCACAAAT
Diptericin B (DiptB)	AGGATTCGATCTGAGCCTCAACGG	TGAAGGTATACACTCCACCGGCTC
Drosocin (Dro)	GCTGCTTGCTTGCGTTT	TGAGTCAGGTGATCCTCGAT
Drosomycin (Drs)	AGTACTTGTTCGCCCTCTTCGCTG	CCTTGTATCTTCCGGACAGGCAGT
Metchnikowin (Mtk)	CATCAATCAATTCCCGCCACCGAG	AAATGGGTCCCTGGTGACGATGAG
Ribosomal protein L32 (RpL32)	GACGCTTCAAGGGACAGTATCTG	AAACGCGGTTCTGCATGAG
Spätzle (Spz)	ACTTCGCCGCCAACTTT	AGGAATTCTGCACCACGTC
Spz3	CAGCAGGATCAGCATGAGAA	TCAGCTGGATGGAACTTTGG
Spz4	CCACTGCTCCGGAAATAGTTAG	GTGGCGCAGTTCCTTTAGTA
Spz5	AGAAGCGTTCGAGGACAAAG	AGTTTCCGCGGCTATTCAG
Spz6	GTTATTACGCCTTCGTCGAGTC	GTCCTTGCACTCCGCATTTA
TAF1	GGCCAAGTCAAATGATGCATCTAGTCCC	CAGCTTCCGATCCGCATCCTTTG
Turandot A (TotA)	CGAGCATACGGATGCCATTA	CCTTCACACCTGGAGATACAATC
TotC	CTAATCGATGGAGTTCCGAGTC	TGCCACCGAGTTCTTCAAA
TotM	GAGGCGTTACAAGGAGGAAA	GACTCCCTCAGAGGCAATTT
TotX	CAGAGAATACCGGGCAGTTT	ATATACCGGGTTCCGACTCT
Unpaired 1 (Upd1)	ACCACAAGAAGCAGCAGAG	GGCTTGGATACCTCGTTGAA
Upd2	CGTCATCGTCATCCTCATCATC	GCTACTCTCGCTGAGACTCATA
Upd3	GCTGACCTTCCAGCAGAAAT	TGCTGTGCGTTTCGTTCA
